# Management of Soft Tissue Defects Around Single Implants: A Systematic Review of the Literature

**DOI:** 10.1002/cre2.70003

**Published:** 2024-11-04

**Authors:** Haithem Moussa, Wafa Nasri, Rania Gargouri, Afif Bouslema

**Affiliations:** ^1^ Department of Periodontology, Faculty of Dental Medicine of Monastir University of Monastir Monastir Tunisia; ^2^ Oral Health and Oro‐Facial Rehabilitation Laboratory Research LR12ES11 Monastir Tunisia; ^3^ Department of Stomatology University Hospital Sahloul Sousse Tunisia

**Keywords:** connective tissue, dental esthetics, patient‐reported outcome measures, single‐tooth dental implants, tissue transplantation

## Abstract

**Objectives:**

The aim of this systematic review was to assess the effectiveness of the available techniques for the management of peri‐implant soft tissue defects around single implants in the anterior region.

**Material and Methods:**

A comprehensive search was conducted in PubMed (MEDLINE), Web of Science (all databases), and Cochrane, using keywords and MeSH terms related to the topic. This systematic review included prospective interventional studies with a minimum of 10 patients and at least 6 months of follow‐up.

**Results:**

A total of 13 articles were included, with eight focusing on outcomes related to buccal soft tissue dehiscence coverage procedures and the remaining five investigating interventions aimed at augmenting soft tissue thickness. Coronally advanced flap in combination with connective tissue graft was the most effective technique for buccal soft tissue dehiscence coverage in the medium and long term. In terms of increasing soft tissue thickness, both connective tissue graft and acellular dermal matrix demonstrated satisfactory short‐term outcomes; however, their long‐term efficacy remains unclear.

**Conclusions:**

Soft tissue augmentation procedures resulted in satisfactory outcomes, in terms of buccal soft tissue dehiscence coverage and soft tissue thickness increase, around single implants in the esthetic area. Peri‐implant plastic surgery has improved both the esthetic appearance and quality of life of patients.

**PROSPERO Registration Code:**

CRD42023398424

## Clinical Relevance

The presence of peri‐implant soft tissue defects, especially in the esthetic zone, can significantly impact the overall success of implant therapy. There is limited evidence on the effectiveness of various surgical procedures for treating these defects. Thus, an evidence‐based approach is crucial. In this review, different surgical techniques employed to address these defects were compared to recommend the most effective treatment.

## 1 Introduction

Implant therapy has become a valid treatment option for replacing missing teeth, with a high long‐term survival rate (Howe, Keys, and Richards 2019). Over the last decade, the focus of the scientific community has shifted from the functional stability of the implant to its esthetic appearance, which has become an important parameter for clinical success in contemporary oral implantology (Cosyn et al. 2017; Belser, Buser, and Higginbottom 2004).

Esthetic complications are essentially the manifestation of peri‐implant soft tissue defects that have occurred following implant placement (Fürhauser et al. 2005; Jung, Heitz‐Mayfield, and Schwarz 2018). These defects can dramatically impact patients' satisfaction and negatively affect their quality of life (QoL) by inducing feelings of anxiety while smiling, socializing, and speaking in public (Wang et al. 2021; Roccuzzo et al. 2014). Moreover, soft tissue esthetic complications have been linked to greater plaque accumulation, thus potentially jeopardizing the long‐term maintenance of peri‐implant health (Thoma et al. 2022, 2018). Recent findings from the most recent ITI Consensus Conference have extensively analyzed and debated soft tissue augmentation around dental implants. It was concluded that STA provided favorable esthetic outcomes and stable peri‐implant health in the medium and long term (Jensen et al. 2023; Stefanini et al. 2023).

Various soft tissue deficiencies and defects may develop around dental implants (Thoma et al. 2022; Gamborena and Avila‐Ortiz 2021; Mancini et al. 2023), which can be categorized in the following paragraphs.

### 1.1 Lack of Keratinized Mucosa

Peri‐implant keratinized mucosa is the band of keratinized soft tissue that runs in an apico‐coronal direction from the mucosal margin to the mucosal junction (Avila‐Ortiz et al. 2020). The prevalence of the absence of an appropriate band of attached and keratinized tissue has been reported to be 30.4% of the implants placed in the anterior maxilla (Ladwein et al. 2015).

The role of sufficient keratinized mucosa width (KMW) around dental implants is still controversial. Despite the controversies that exist, the presence of keratinized tissue around implants may be beneficial for improving esthetics (Bonino et al. 2018), facilitating effective plaque control and promoting long‐term peri‐implant health (Kim et al. 2009). A recent consensus report from the DGI/SEPA/Osteology Workshop (Sanz et al. 2022) stated that “Reduced KT width is associated with an increased prevalence of peri‐implantitis, biofilm accumulation, soft‐tissue inflammation, mucosal recession, marginal bone loss, and greater patient discomfort.”

### 1.2 Insufficient Soft Tissue Thickness (STT)

Peri‐implant STT is the horizontal dimension of the peri‐implant mucosa, which may or may not be keratinized.

From an esthetic standpoint, studies have demonstrated that a threshold value of 2 mm mucosal thickness is advisable to avoid an unfavorable shine‐through effect of the underlying material (Bienz et al. 2022). Moreover, thick peri‐implant tissue is associated with better esthetic outcomes, improved papilla score, less recession over time, and higher patient satisfaction (Bienz et al. 2022).

From a biological standpoint, soft tissue grafting to increase mucosal thickness results in more favorable peri‐implant health with significantly less marginal bone loss (Thoma et al. 2018).

### 1.3 Buccal Soft Tissue Dehiscence (BSTD)

Peri‐implant BSTD is defined as the apical migration of the mucosal margin, revealing the greyish color of the implant components or increasing the crown height compared with a contralateral site (Sanz‐Martín et al. 2022). This apical displacement of the peri‐implant mucosal margin has been defined throughout the literature using several terms, including mucosal recession, midfacial recession, soft‐tissue recession, mucosal dehiscence, soft‐tissue deficiency, soft‐tissue defect, and marginal mucosa defects (Gamborena and Avila‐Ortiz 2021; Zucchelli et al. 2019).

BSTDs are common findings in the esthetic region with a prevalence of 56.8% (Tavelli et al. 2022) and up to 64% in case of immediate implant placement (Cosyn, Hooghe, and De Bruyn 2012).

The etiology of BSTD may be related to various factors such as the presence of an adjacent implant, an increased time in function of the implant, a buccal bone dehiscence, insufficient amount of keratinized mucosa, thin phenotype, and buccally positioned implant (Tavelli et al. 2022; Cosyn, Sabzevar, and De Bruyn 2012).

Along with the unpleasant outcome, BSTD has also been associated with plaque accumulation and subsequent initiation and/or progression of peri‐ implant disease (Isler et al. 2019).

Therefore, the aim of the present systematic review is to evaluate the effectiveness of the available procedures in the treatment of peri‐implant soft tissue defects/dehiscences around single implants in the esthetic area. Additionally, the review aims to evaluate the esthetic outcomes and PROMs associated with these procedures.

## 2 Materials and Methods

### 2.1 Protocol Development and Registration

The present review was designed and reported according to the guidelines of the Preferred Reporting Items for Systematic reviews and Meta‐Analyses: The (PRISMA) 2020 statement (Page, McKenzie, et al. 2021; Page, Moher, et al. 2021). The review protocol was registered in the International Prospective Register of Systematic Reviews (PROSPERO) database (http://www.crd.york.ac.uk/PROSPERO).

### 2.2 Focused Question

This review was conducted in order to answer the following question: What is the most effective procedure to treat soft tissue defects around single implants in the anterior area?

### 2.3 PICOS Framework

For the above‐mentioned focused question, the following (PICOS) framework (Stillwell et al. 2010) were used to guide the inclusion and exclusion of studies: The population (P) includes periodontally and systemically healthy adults (≥ 18 years) with a single‐tooth implant restoration in the anterior region (non‐molar site) exhibiting soft tissue defects such as lack of keratinized mucosa, thin peri‐implant mucosa, peri‐implant soft tissue recession, or any combination thereof. The intervention (I) involves any surgical procedure aimed at treating the soft tissue defect, with or without grafting material. The comparison (C) assesses the impact of the procedure in terms of defect resolution compared to baseline and between different procedures. The primary outcomes (O) include complete BSTD coverage, mean BSTD coverage, KMW gain, and STT changes. The secondary outcomes include esthetic evaluation and PROMs, such as morbidity, satisfaction, self‐reported esthetics, willingness to retreat, and oral health‐related QoL. The study design (S) encompasses prospective interventional human studies, including randomized controlled clinical trials (RCTs), controlled clinical trials (CCTs), cohort studies, case‐control studies, and case series.

### 2.4 Eligibility Criteria

#### 2.4.1 Inclusion Criteria

➢ Osseointegrated loaded (regardless of the period of loading) or uncovered (with a minimum of 2 months of soft tissue healing) single‐tooth implants.
➢ Studies should report at least one primary outcome.
➢ Studies with at least 6 months of follow‐up and a minimum of 10 patients.
➢ Publication's recency: Last 10 years (2013–2024).

#### 2.4.2 Exclusion Criteria

➢ Implants with a diagnosis of peri‐implantitis. Implants in the posterior region.
➢ Multiple adjacent implants present soft tissue defects.
➢ Retrospective clinical studies, case reports, in vitro, or animal studies.
➢ Intervention at implant placement or at second‐stage surgery.
➢ Intervention around teeth.
➢ Systemic conditions that can impair oral wound healing (e.g., uncontrolled diabetes).
➢ Previous soft tissue defect treatment at the implant site within the past 6 months.

### 2.5 Information Sources and Search Strategy

See Supporting Information S1: Tables S1–3.

### 2.6 Data Collection and Management

See Supporting Information S1: Appendix.

### 2.7 Quality Assessment and Risk of Bias

The risk of bias for the included studies was assessed independently and in duplicate by the two reviewers. For RCTs, it was performed according to the recommended approach by the Cochrane Collaboration Group (Higgins et al. 2011). For non‐randomized cohort studies included in the qualitative analysis, the ROBINS‐I tool (Sterne et al. 2016) was used to determine the potential risk of bias. For the case series, the Joanna Briggs Institute Critical Appraisal tool (Munn et al. 2020) was utilized for quality assessment. Any disagreement was discussed between the same authors. However, no study was excluded based on the risk of bias within a study.

### 2.8 Data Analysis

Due to the expected heterogeneity and limited sample size per outcome, the results of the current systematic review were expressed qualitatively and without quantitative analysis. The descriptive analysis was performed per type of soft tissue defect, measured in each study, and presented as stated in the original report.

Inter‐rater agreement following full‐text assessment was calculated via kappa statistics.

## 3 Results

### 3.1 Search Results and Study Selection

**Figure 1:**
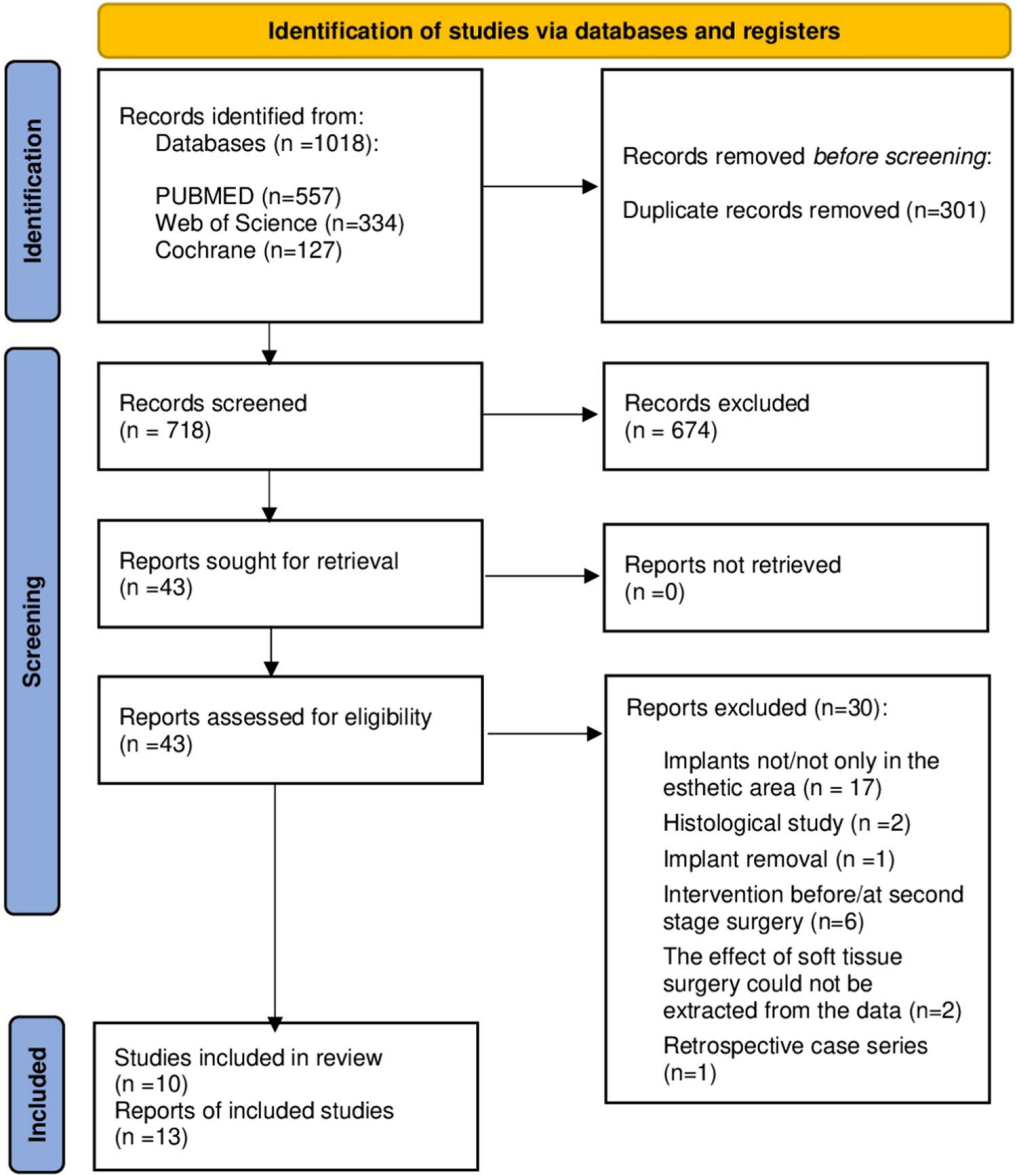
PRISMA flowchart.

The literature search process is shown in Figure 1. The global search initially yielded 1208 records in total. Following the removal of duplicates using the citation manager “Zotero software,” 907 records were screened based on titles and abstracts. Forty‐three articles were sought for retrieval and then fully assessed for eligibility. Based on the predetermined inclusion criteria, 13 articles were included in the qualitative analysis (Roccuzzo et al. 2014; De Bruyckere et al. 2015; Eghbali et al. 2018; Hosseini, Worsaae, and Gotfredsen 2020; Anderson et al. 2014; Roccuzzo et al. 2019; Stefanini, Rendon, and Zucchelli 2020; Tavelli, Majzoub, et al. 2023; Tavelli, Zucchelli, et al. 2023; Zucchelli et al. 2013, 2018; Roccuzzo et al. 2024). The remaining 30 articles were excluded. The manual search yielded a total of three articles. One of them could not be retrieved (Kosinski 2013), and the remaining two were excluded after full‐text screening (Chacón R and Retana 2022; Urban, Klokkevold, and Takei 2017). The reasons for exclusion are detailed in Supporting Information S1: Table S4. The inter‐rater reliability in the screening and inclusion process, as measured by Cohen, corresponded to 0.88 and 0.90 for assessment of titles and abstracts and full‐text evaluation, respectively. These values indicate the strong agreement between the raters in both stages of the screening process (McHugh 2012).

### 3.2 Description of Studies

The overall characteristics of the included studies are presented in Table 1. None of the included studies investigated the augmentation of KMW. Among the 13 included studies, eight reported interventions to cover BSTD (Roccuzzo et al. 2014, 2019, 2024; Anderson et al. 2014; Tavelli, Majzoub, et al. 2023; Tavelli, Zucchelli, et al. 2023; Zucchelli et al. 2013, 2018). The remaining five studies examined procedures to increase STT (De Bruyckere et al. 2015; Eghbali et al. 2018; Hosseini, Worsaae, and Gotfredsen 2020; Stefanini, Rendon, and Zucchelli 2020; Eghbali et al. 2016).

**Table 1:** Characteristics of the included studies.

Author (year)	Study design	Number of centers, country, setting, funding	Sample size	Population characteristics: Age (year), number of male/female, number of smokers	Number of implants and localization	Soft tissue defect	Timing of the intervention	Intervention	Follow‐up (months)
Zucchelli et al. (2013)	Prospective case series	Single center, Italy, University, self‐ supported	20	26–53 6/14 NA	20 esthetic area (from first right premolar to first left premolar)	BSTD with no interproximal attachment loss for the teeth neighboring the implant	Loaded implants with more than 2 years of loading	Surgical‐prosthetic approach: ‐ Restoration removal, abutment reduced and polished if necessary to create an adequate finishing line, and provisional restoration ‐ CAF + CTG 1 month later	12
Zucchelli et al. (2018)	Prospective case series	Single center, Italy, University, self‐ supported	20	NA NA NA	19 Esthetic area (from first right premolar to first left premolar)	BSTD with no interproximal attachment loss for the teeth neighboring the implant	Loaded implants with more than 2 years of loading	Surgical‐prosthetic approach: ‐ Restoration removal, abutment reduced and polished if necessary to create an adequate finishing line, and provisional restoration ‐ CAF + CTG 1 month later	60
Roccuzzo et al. (2014)	Prospective case series	Single center, Italy, private practice, self‐ supported	16	53.1 ± 11.7 3/13 3	16 Maxillary area (from the first right molar to the second left premolar)	BSTD with no interproximal attachment loss and/or adjacent papillae recession	Loaded implants with 14–120 months of loading	Split‐thickness envelope CAF + CTG	12
Roccuzzo et al. (2019)	Prospective case series	Single center, Italy, private practice, self‐ supported	16	NA 2/11 2	13 Maxillary area (from the first right molar to the second left premolar)	BSTD with no interproximal attachment loss and/or adjacent papillae recession	Loaded implants with 14–120 months of loading	Split‐thickness envelope CAF + CTG	60
Roccuzzo et al. (2024)	Prospective case series	Single center, Italy, private practice, self‐ supported	16	NA 2/11 2	13 Maxillary area (from the first right molar to the second left premolar)	BSTD with no interproximal attachment loss and/or adjacent papillae recession	Loaded implants with 14–120 months of loading	Split‐thickness envelope CAF + CTG	120
Anderson et al. (2014)	Two‐arm RCT	Single center, USA, University, sponsored	Test: 6 Control: 7	NA NA NA	Test: 6 Control: 7 Maxillary non‐molar implants	Implant with failing pink esthetic profile (2 mm soft tissue concavity or 2 mm recession)	Loaded implants	Test group: CAF + ADM Control group: CAF + SCTG	6
Eghbali et al. (2016)	Prospective case series	Single center, Belgium, University, self‐ supported	10	52 3/7 0	10 Anterior maxilla (from the second right premolar to the second left premolar)	Implants demonstrating alveolar process deficiency	Uncovered implants (3 months following implant installation)	Split‐thickness envelope (pouch) + GTC (harvested from the palate using trap door approach)	9
De Bruyckere et al. (2015)	Prospective case series	Single center, Belgium, University, sponsored	37	38 19/18 0	37 Anterior maxilla (from the second right premolar to the second left premolar)	Implants demonstrating alveolar process deficiency (buccolingual loss of tissue with normal apicocoronal ridge height)	Uncovered implants (3 months following implant installation)	‐ Provisional screw‐retained crown with a concave buccal emergence profile ‐ Split‐thickness envelope (pouch) + CTG	9
Eghbali et al. (2018)	Prospective case series	Single center, Belgium, University, sponsored	37	38 19/18 0	37 Anterior maxilla (from the second right premolar to the second left premolar)	Implants demonstrating alveolar process deficiency (buccolingual loss of tissue with normal apicocoronal ridge height)	Uncovered implants (3 months following one‐stage implant installation)	‐ Connection of provisional screw‐ retained crown with a concave buccal emergence profile ‐ Split‐thickness envelope (pouch) + CTG (harvested from the palate with single incision approach)	60
Hosseini, Worsaae, and Gotfredsen (2020)	CCT	Single center, Denmark, University, self‐ supported	19	22 8/11 NA	33 Test: 10 Control: 23 Anterior maxilla	Recession of the mucosa around the healing abutment, thin gingival phenotype with dark discoloration of the facial mucosa, remaining defect or a combination thereof	Uncovered implants (2–3 months following one‐stage implant installation)	Test group: ‐ Split‐thickness envelope CAF + CTG Control group: ‐ NSTG	60
Stefanini, Rendon, and Zucchelli (2020)	Prospective case series	Single center, Italy, University, sponsored	10	48.1 4/6 NA	10 Esthetic area (from second premolar to contralateral second premolar)	Buccal soft tissue deficiency with unesthetic appearance or discomfort caused by food impaction	Loaded implants	‐ Restoration was replaced with a narrow screw‐retained provisional crown 2 months before the intervention ‐ CAF + ADM	12
Tavelli, Zucchelli, et al. (2023)	Prospective case series	Single center, University, USA, self‐ supported	10	52.8 ± 13.9 3/7 NA	10 Anterior maxilla (from canine to contralateral canine)	BSTD with at least one adjacent tooth with interproximal attachment loss	Loaded implants with mean loading time 8.6 ± 2.7 years	Vertical soft tissue augmentation with implant submersion: ‐ Crown removal + CAF + horizontal augmentation with CTG + vertical augmentation at the level of implant site with a second CTG + PRF applied over the CTGs prior to flap closure	12
Tavelli, Majzoub, et al. (2023)	Two‐arm RCT	Single center, USA, sponsored	28	47 ± 12.1 12/16 2	28 Group 1: 14 Group 2: 14 Non‐molar site	Class II PSTD Subclass a or b	Loaded implants with loading time 12 months	Group 1: ‐ Split‐thickness envelope CAF + CTG Group 2: ‐ Tunnel + CTG	12

Abbreviations: ADM, acellular dermal matrix; BSTD, buccal soft tissue dehiscence; CAF, coronally advanced flap; CCT, controlled clinical trial; CTG, connective tissue graft; NA, not available; NSTG, no soft tissue graft; PRF, platelet‐rich fibrin, RCT, randomized controlled trial; SCTG, sub‐epithelial connective tissue graft.

#### 3.2.1 BSTD

Two studies were designed as two‐arm RCTs (Anderson et al. 2014; Tavelli, Majzoub, et al. 2023), and the other six were prospective case series (Roccuzzo et al. 2014, 2019, 2024; Tavelli, Zucchelli, et al. 2023; Zucchelli et al. 2013, 2018). Three reports (Roccuzzo et al. 2019, 2024; Zucchelli et al. 2018) presented an extended follow‐up of previous studies (Roccuzzo et al. 2014; Zucchelli et al. 2013). The included studies provided data for 87 patients, each with one implant. The sample size within studies ranged from 10 to 28 (Tavelli, Majzoub, et al. 2023; Tavelli, Zucchelli, et al. 2023). The follow‐up period varied among the included studies, ranging from 6 months to 10 years (Anderson et al. 2014; Roccuzzo et al. 2024).

Regarding the clinical attachment level (CAL) status of the teeth neighboring the implants, no CAL loss was reported in six studies (Roccuzzo et al. 2014, 2019, 2024; Tavelli, Majzoub, et al. 2023; Zucchelli et al. 2013, 2018), one study investigated BSTD with at least one adjacent tooth with interproximal attachment loss (Tavelli, Zucchelli, et al. 2023), and one study did not report the CAL of the adjacent teeth (Anderson et al. 2014).

With respect to the interventions, coronally advanced flap (CAF) with connective tissue graft (CTG) was used in five studies (Roccuzzo et al. 2014, 2019; Zucchelli et al. 2013, 2018; Roccuzzo et al. 2024), in one group of the two‐arm RCT by Tavelli, Majzoub, et al. (2023), and in the control group of the two‐arm RCT by Anderson et al. (2014). Additionally, Zucchelli et al. (Zucchelli et al. 2013, 2018) conducted crown removal, abutment adjustment, and provisional restorations 1 month before the intervention. Tunnel technique (TUN) with CTG was performed for the second group of the two‐arm RCT by Tavelli, Majzoub, et al. (2023), and CAF with acellular dermal matrix (ADM) was used for the test group of the two‐arm RCT by Anderson et al. (2014). The remaining study (Tavelli, Zucchelli, et al. 2023) described vertical soft tissue augmentation with implant submersion by removing the crown, CAF with two CTG, one at the horizontal aspect of the defect and the other vertical over the top of the implant, and finally, patelet‐rich fibrin (PRF) was applied over the CTG's and the flap closed.

Except for one study that utilized sub‐epithelial CTG (SCTG) without specifying the harvesting technique (Anderson et al. 2014), all studies used de‐epithelialized free gingival graft (FGG); however, the donor site differed. The palate was employed in three studies (Tavelli, Majzoub, et al. 2023; Zucchelli et al. 2013, 2018), the tuberosity in two studies (Roccuzzo et al. 2014, 2019), and either the lateral palate or the tuberosity in one study (Tavelli, Zucchelli, et al. 2023). All the interventions were performed around osseointegrated loaded implants.

#### 3.2.2 Soft Tissue Thickness

One study was designed as CCT (Hosseini, Worsaae, and Gotfredsen 2020), and the other four were prospective case series (De Bruyckere et al. 2015; Eghbali et al. 2016, 2018; Stefanini, Rendon, and Zucchelli 2020). One report (Eghbali et al. 2018) was an extended follow‐up of a previous study (De Bruyckere et al. 2015). The included investigations provided data for 76 patients and 90 implants. Only one study investigated more than one implant in a single patient (Hosseini, Worsaae, and Gotfredsen 2020). The sample size within studies ranged from 10 to 37 patients. The follow‐up period was 9 months in two studies (De Bruyckere et al. 2015; Eghbali et al. 2016), 12 months in one study (Stefanini, Rendon, and Zucchelli 2020), and 60 months in two studies (Eghbali et al. 2018; Hosseini, Worsaae, and Gotfredsen 2020).

Split‐thickness envelope with CTG was performed in three studies (De Bruyckere et al. 2015; Eghbali et al. 2016, 2018), split‐thickness CAF with CTG was utilized in the test group of the CCT (Hosseini, Worsaae, and Gotfredsen 2020), and CAF with porcine‐derived ADM in one study (Stefanini, Rendon, and Zucchelli 2020).

Additionally, a provisional screw‐retained crown with a concave buccal emergence profile was connected before the procedure in two studies (De Bruyckere et al. 2015; Eghbali et al. 2018), and restoration was replaced with a narrow screw‐retained provisional crown 2 months before the intervention in one study (Stefanini, Rendon, and Zucchelli 2020).

The CTG harvesting techniques varied across the studies included. In one study (Eghbali et al. 2016), the CTG was harvested using the trap door technique. Two studies (De Bruyckere et al. 2015; Eghbali et al. 2018) utilized a single incision approach for CTG harvesting. In another study (Hosseini, Worsaae, and Gotfredsen 2020), the CTG was obtained through the de‐epithelialized FGG technique.

### 3.3 Effects of Intervention

#### 3.3.1 BSTD

The main results are summarized in Table 2.

**Table 2:** Primary and secondary outcomes of the included studies for BSTD coverage.

Author (year)	Primary outcomes						Secondary outcomes				
	Mean BSTD at baseline (mm)	Mean BSTD at last follow‐up (mm)	Mean BSTD coverage (mm and/or %)	% of complete BSTD coverage	KMW gain (mm)	STT increase (mm)	Professiona l esthetic assessment (index, mean value)	PROMs			
								Patient esthetic evaluation	Morbidity	QoL	Other PROMs
Zucchelli et al. (2013)	2.72 ± 0.68	0.10 ± 0.44	2.62 ± 0.81 96.3%	75%	0.57 ± 0.41	1.58 ± 0.21	PES/WES 17.7 ± 1.17	VAS (0–10) 8.0; (95% CI 8–10)	NA	NA	NA
Zucchelli et al. (2018)	3.0 (quartiles, 2.0–3.0)	0.0 (quartiles, 0.0–0.0)	3.0 (quartiles, 2.0–3.0) 99.2%	79%	1.0 (quartiles, 0.5–2.0)	1.8 (quartiles, 1.60– 2.10)	PES/WES 17.63 ± 0.96	VAS (0–10) 8.95 ± 0.91	NA	NA	NA
Roccuzzo et al. (2014)	1.9 ± 0.7	0.2 ± 0.3	89.7 ± 12.9%	56.3%	NA	NA	VAS (0–10) 8.5 ± 0.3	NA	NA	NA	NA
Roccuzzo et al. (2019)	1.9 ± 0.7	0.2 ± 0.3	86.0 ± 19%	62%	NA	NA	VAS (0–10) 8.1 ± 0.9	VAS (0–10) 9.5 ± 0.8	NA	NA	NA
Roccuzzo et al. (2024)	1.9 ± 0.7	0.2 ± 0.2	89.6 ± 17.1%	58.3%	NA	NA	VAS (0–10) 8.5 ± 0.9	VAS (0–10) 9.5 ± 0.8	NA	NA	NA
Anderson et al. (2014)	Test 1.2 Control 0.7	Test 0.83 Control 0.43	Test 0.37 28% Control 0.27 40%	NA	NA	Test 1.75 mm Control 1.00 mm	CEI significantly improved over time in both groups NSSD between groups	Patient‐ modified CEI NSSD change over time in both groups NSSD between groups	VAS (0–10) Test: 9.34 (2 weeks) 4.00 (6 weeks) 3.66 (3 months) 0.34 (6 months) Control 8.86 (2 weeks) 0 (6 weeks) 0 (3 months) 0 (6 months)	5‐point QoL scale A slight reduction in QoL of the control group, whereas a slight increase in QoL of the test group over time No significant differences were found between groups	Wound healing index test 2.50 ± 0.54 (2 weeks) Control 1.57 ± 0.53 2 (weeks) Both groups improved over time Medication use both groups showed a reduction in medication use and strength over time greater quantity and strength in the control group
Tavelli, Zucchelli, et al. (2023)	2.60 ± 0.61	0.35 ± 0.47	2.25 ± 0.82 85.14 ± 21.11%	NA	1.15 ± 1.06	1.58 ± 0.61	IDES 6.90 ± 2.33	VAS (0–10) 8.83	VAS (0–10) 2.63 (2 weeks)	NA	Willingness for retreatment All the treated subjects stated that they would be available for retreatment, if needed
Tavelli, Majzoub, et al. (2023)	CAF 2.46 ± 0.87 Tunnel 2.36 ± 0.46	CAF 0.25 ± 0.47 Tunnel 1.00 ± 0.88	CAF 90.23 ± 19.85% Tunnel 59.76 ± 34.94%	CAF 71.4% Tunnel 28.6%	CAF 2.57 ± 0.90 Tunnel 1.57 ± 1.05	CAF 1.48 ± 0.47 Tunnel 1.02 ± 0.49	IDES CAF 7.00 2.45 (6 months) 7.29 2.58 (12 mo) Tunnel 4.93 2.53 (6 months) 4.86 2.41 (12 months)	VAS (0–100) CAF 74.4 ± 16.0 (12 months) Tunnel 43.4 ± 13.5 (12 months)	VAS (0–100) CAF 18.9 ± 9.5 Tunnel 14.2 ± 8.4	VAS (0–100) CAF 41.3 ± 23.5 (baseline) 5.83 ± 4.01 (12 months) Tunnel 37.9 ± 22.1 (baseline) 19.26 ± 6.80 (12 months)	Time to recovery (VAS < 10) CAF 7.4 ± 3.9 days Tunnel 6.4 ± 3.7 days

Abbreviations: BSTD, buccal soft tissue dehiscence; CAF, coronally advanced flap; CI, confidence interval; CEI, complex esthetic index; IDES, implant dehiscence coverage esthetic score; KMW, keratinized mucosa width; NA, not available; NSSD, no statistically significant difference; PES/WES, pink esthetic score/white esthetic score; PROMs, patient‐reported outcome measures; QoL, quality of life; STT, soft tissue thickness; VAS, visual analogue scale.

##### 3.3.1.1 Primary Outcomes

###### 3.3.1.1.1 BSTD Coverage

**Figure 2:**
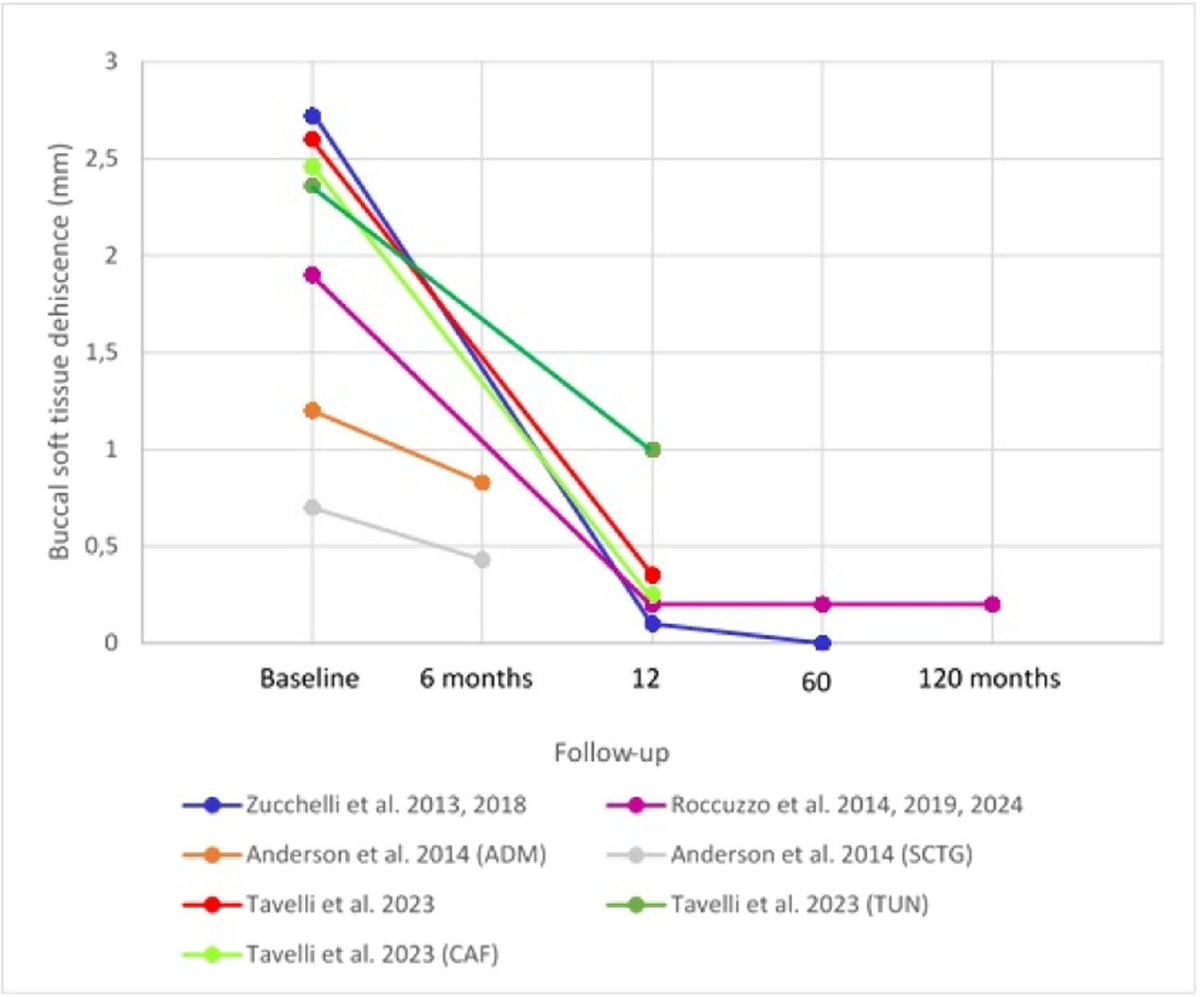
Buccal soft tissue dehiscence change over time.

The spaghetti plot in Figure 2 illustrates the change in BSTD in millimeters throughout the follow‐up periods of the included studies. The highest percentage of mean BSTD coverage was reported by Zucchelli et al. with up to 99.2% at 5‐year follow‐up (Zucchelli et al. 2018) and 96.3% after 1 year (Zucchelli et al. 2013). In this study, the authors obtained a mean BSTD reduction of 2.62 mm after 1 year (Zucchelli et al. 2013), and greater (but not statistically significant) reduction after 5 years, with a mean value of 3.0 mm (Zucchelli et al. 2018). Roccuzzo et al. (2014) reported a mean BSTD coverage of 89.7% at 1 year, which remained stable throughout the 5‐year follow‐up, and the rest of the observation period, with a mean BSTD coverage of 89.6% at the 10‐year examination (Roccuzzo et al. 2019, 2024). Almost the same percentage, 85.14%, was reported by Tavelli, Zucchelli, et al. (2023). However, the mean BSTD coverage was higher with a value of 2.25 mm after 1 year. In their two‐arm RCT, Tavelli, Majzoub, et al. (2023) found significantly (*p* = 0.03) higher mean BSTD coverage in the CAF group (90.23%) compared to the TUN group (59.76%). Anderson et al. (2014) achieved the lowest mean BSTD coverage, with up to 40% in the CTG group and about 28% in the ADM group. The mean BSTD reduction measured in the control and test groups was only 0.27 and 0.37 mm, respectively.

###### 3.3.1.1.2 KMW Gain

KMW gain was assessed only in four studies (Tavelli, Majzoub, et al. 2023; Tavelli, Zucchelli, et al. 2023; Zucchelli et al. 2013, 2018). Zucchelli et al. reported a 0.57 mm increase in KMW after 1 year (Zucchelli et al. 2013) and a 1 mm increase after 5‐year follow‐up (Zucchelli et al. 2018). Tavelli, Zucchelli, et al. (2023) reported a mean KMW gain of 1.15 mm in their prospective study. The KMW gain in the CAF group of the two‐arm RCT by Tavelli, Majzoub, et al. (2023) was about 2.57 mm, which was significantly (*p* = 0.01) higher than the TUN group's gain of about 1.57 mm.

###### 3.3.1.1.3 STT Increase

STT increase was not reported in two studies (Roccuzzo et al. 2014, 2019). The STT increased by 1.57 mm after 1 year in two studies (Tavelli, Zucchelli, et al. 2023; Zucchelli et al. 2013). Zucchelli et al. reported a 1.8 mm increase in STT at 5‐year follow‐up (Zucchelli et al. 2018). Anderson et al. (2014) found that soft tissue thickening was about 1.75 mm in the ADM group and 1 mm in the CTG group after 6 months. Tavelli, Majzoub, et al. (2023) reported STT increase by 1.48 mm in the CAF group and by 1.02 mm in the TUN group after 1 year.

##### 3.3.1.2 Secondary Outcomes

###### 3.3.1.2.1 Professional Esthetic Assessment

Professional esthetic evaluation was reported in all the studies. Zucchelli et al. (2013, 2018) used PES/WES and reported a significant (*p* < 0.001) improvement, with a mean value of 17.7 after 1 year. This value remained stable after 5 years (*p* > 0.005). Roccuzzo et al. (2014) reported a statistically significant (*p* < 0.0001) improvement in the VAS, from 3.6 at baseline to 8.8 at the 1‐year follow‐up. This improvement remained stable during the 5‐year follow‐up (VAS = 8.1) and the 10‐year observation period (VAS = 8.5) (Roccuzzo et al. 2019, 2024). Using the CEI, Anderson et al. (2014) found a significant (*p* = 0.001) improvement over time in both the control and test groups, without a statistically significant (*p* = 0.073) difference between them. IDES was employed in two studies (Tavelli, Majzoub, et al. 2023; Tavelli, Zucchelli, et al. 2023). Tavelli et al. reported a mean value of 6.9 in one study (Tavelli, Zucchelli, et al. 2023), while the same author found that CAF resulted in a significant (*p* = 0.01) superior esthetic outcome with a score of 7.29 when compared to TUN with a score of 4.86 in the other study (Tavelli, Majzoub, et al. 2023).

###### 3.3.1.2.2 Patients' Esthetic Evaluation

Except for Andreson et al. (2014), who used a patient‐modified CEI, all studies employed the VAS to evaluate the patient‐reported esthetic. Anderson et al. (2014) did not find a statistically significant change over time (*p* = 0.204) and between the two groups (*p* = 0.086). Zucchelli et al. (2013) and Tavelli, Zucchelli, et al. (2023) reported a substantial improvement in VAS scores, with a mean score of 8 and 8.8, respectively, after 1 year. The result achieved by Zucchelli et al. remained stable throughout the 5‐year follow‐up period (Zucchelli et al. 2018). Roccuzzo et al. (2019) investigated patient‐reported satisfaction at the 5‐year follow‐up and documented a mean VAS score of 9.5, which remained stable for the rest of the 10‐year observation period (Roccuzzo et al. 2024). In the two‐arm RCT, Tavelli, Majzoub, et al. (2023) stated that the perceived esthetic improved significantly from baseline to 12 months in both groups (*p* < 0.01) and was in favor of CAF (74.4 VAS) over TUN (43.4 VAS) (*p* = 0.03).

###### 3.3.1.2.3 Morbidity

Pain perception was reported in three studies (Anderson et al. 2014; Tavelli, Majzoub, et al. 2023; Tavelli, Zucchelli, et al. 2023). Anderson et al. (2014) found that similar levels of pain were reported at 2 weeks postoperatively in both the control and test groups, with VAS (0–10) values of 8.86 and 9.34, respectively. However, pain progressively disappeared over 6 months in the test group, while it was only observed for 2 weeks in the control group. Tavelli et al (Tavelli, Majzoub, et al. 2023) stated that the overall morbidity rates in the first postoperative week were 18.9 and 14.2 VAS (0–100) in the CAF and TUN groups, respectively, with no significant difference between the two groups in the second postoperative week (*p* > 0.05). The same author reported a mean morbidity of 2.63 VAS (0–10) during the first 2 weeks in the other study (Tavelli, Zucchelli, et al. 2023).

###### 3.3.1.2.4 QoL

The impact of the treatment on the patient's QoL was investigated in two studies (Anderson et al. 2014; Tavelli, Majzoub, et al. 2023). Anderson et al. (2014), using a 5‐point QoL scale, found no significant difference between the test and control groups (*p* = 0.672). Tavelli, Majzoub, et al. (2023) evaluated the QoL in terms of anxiety related to the appearance of the dental implant and its possible negative impact on speaking, socializing, and eating using a VAS (0–100). The author reported a noticeable reduction of anxiety in both CAF and TUN groups (*p* = 0.03).

#### 3.3.2 STT

The main results are summarized in Table 3.

**Table 3:** Primary and secondary outcomes of the included studies for STT augmentation.

Author (year)	Primary outcomes				Secondary outcomes				
	Mean STT at baseline (mm)	Mean STT at last follow‐up (mm)	STT increase (mm)	KMW gain (mm)	Professional esthetic assessment	PROMs			
						Patient esthetic evaluation	Morbidity	QoL	Other PROMs
De Bruyckere et al. (2015)	1.51 (SD = 0.46)	2.50 (SD = 0.56)	0.97 (SD = 0.48)	NA	PES 11.00 (SD = 1,64)	NA	NA	NA	NA
Eghbali et al. (2018)	1.51 (SD = 0.46)	2.42 (SD = 0.63)	0.91 (SD = 0.57)	NA	PES 11.17 (SD = 1.91)	NA	NA	NA	NA
Eghbali et al. (2016)	1.65 (SD = 0.41)	2.48 (SD = 0.30)	0.83	NA	NA	NA	NA	NA	NA
Hosseini, Worsaae, and Gotfredsen (2020)	NA	NA	Control 1 mm: −0.68 (SD = 0.96) 3 mm: −0.46 (SD = 1.01) CTG 1 mm: −0.04 (SD = 1.03) 3 mm: −0.16 (SD = 0.77)	The height of the keratinized mucosa did not alter significantly between different observations or between the test and control group	CIS The discoloration scores were significantly lower in the test group The papilla index scores were not significantly different between the test and control group	NA	NA	NA	NA
Stefanini, Rendon, and Zucchelli (2020)	1.03 ± 0.21	2.23 ± 1.89	1.2 ± 0.18	0.65 ± 0.41	NA	NA	NA	NA	NA

Abbreviations: CIS, Copenhagen index score; CTG, connective tissue graft; KMW, keratinized mucosa width; NA, not available; PES, pink esthetic score; PROMs, patient‐reported outcome measures; QoL, quality of life; SD, standard deviation; STT, soft tissue thickness.

##### 3.3.2.1 Primary Outcomes

###### 3.3.2.1.1 STT Increase

**Figure 3:**
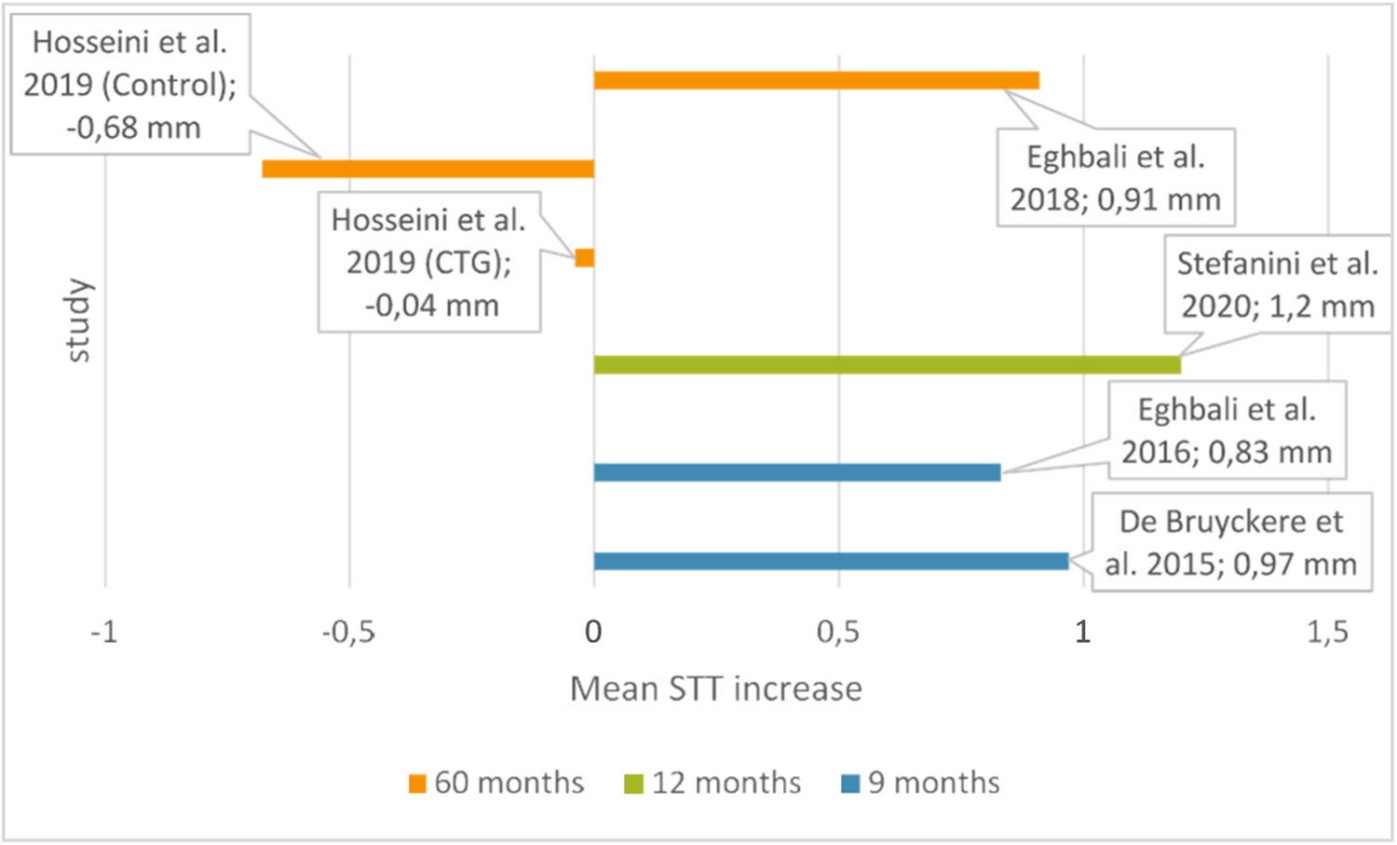
Mean soft tissue thickness changes across studies.

The mean STT change across studies is shown in Figure 3. Except for one (Hosseini, Worsaae, and Gotfredsen 2020), all studies reported increased STT during follow‐up. Stefanini, Rendon, and Zucchelli (2020) recorded the highest STT gain, with a mean increase of 1.2 mm over 1 year. In the study of Eghbali et al. (2016), the STT increased by 0.83 mm after 9 months. De Bruyckere et al. (2015) found that the STT increased by approximately 0.97 mm in 9 months. Furthermore, the authors sorted changes in mucosal thickness by biotype and found that patients with thick biotype had significantly more soft tissue loss at 9 months after CTG (*p* = 0.039). However, the difference was not statistically significant (p 0.290). In an extended follow‐up of the previous study, Eghbali et al. (2018) noticed that the STT remained stable after 5 years, with a mean increase of 0.91 mm at the last follow‐up, corresponding to a relative horizontal stability of 85%.

Interestingly, Hosseini, Worsaae, and Gotfredsen (2020) found that STT did not change significantly from baseline to the 5‐year observation in the test group, with a mean value of −0.04 mm at 1 mm from the crown margin and −0.16 mm at 3 mm (*p* = 0.273). In contrast, STT in the control group decreases significantly from baseline to the 5‐year observation with a mean value of −0.68 mm at 1 mm and −0.46 mm at 3 mm (*p* = 0.032).

###### 3.3.2.1.2 KMW Gain

KMW change was recorded in two studies (Hosseini, Worsaae, and Gotfredsen 2020; Stefanini, Rendon, and Zucchelli 2020). Stefanini, Rendon, and Zucchelli (2020) reported a 0.65 mm increase in the width of the keratinized mucosa after 1 year. Hosseini, Worsaae, and Gotfredsen (2020) reported that the height of the keratinized mucosa did not change significantly between different observations or between the test and control groups (*p* = 0.551).

##### 3.3.2.2 Secondary Outcomes

Esthetic was professionally assessed in three studies (De Bruyckere et al. 2015; Eghbali et al. 2018; Hosseini, Worsaae, and Gotfredsen 2020). Eghbali et al. (2018) reported that the PES score resulted in a mean value of 11.17 after 5 years, with no significant change (*p* = 0.596) compared to the 9‐month outcome (PES = 11.0) (De Bruyckere et al. 2015). Hosseini, Worsaae, and Gotfredsen (2020) used CIS and found that the discoloration scores were significantly lower in the test group (*p* = 0.035), whereas the papilla index scores were not significantly different between the test and control group (*p* = 0.757 for mesial papilla and *p* = 0.9 for distal papilla).

PROMs were not reported in any of the included studies.

### 3.4 Assessment of the Risk of Bias

**Figure 4:**
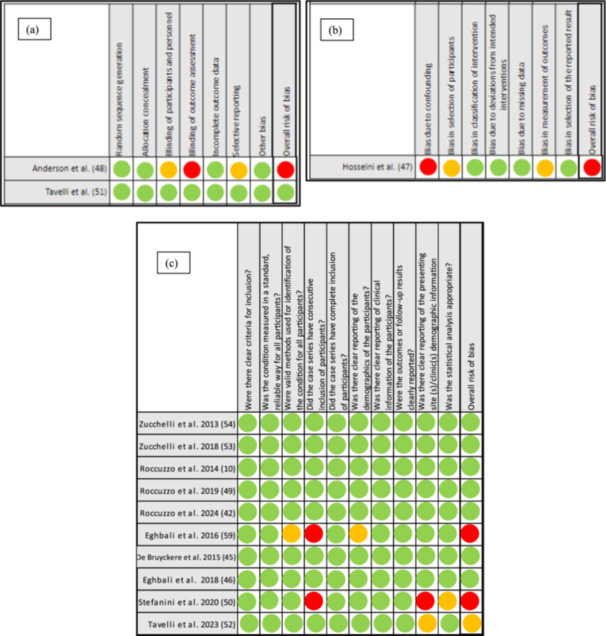
(a) Risk of bias assessment of RCTs. (b) Risk of bias assessment of CCT. (c) Risk of bias assessment of case series.

The quality and risk of bias assessments of included studies are presented in Figure 4. Two of the included studies were RCTs (Anderson et al. 2014; Tavelli, Majzoub, et al. 2023), one of them was considered to have a low risk of bias (Tavelli, Majzoub, et al. 2023), while the other (Anderson et al. 2014) was assigned a high risk of bias. One study was non‐RCT (Hosseini, Worsaae, and Gotfredsen 2020), and the estimated risk of bias was high. The other 10 included studies were prospective case series (Roccuzzo et al. 2014, 2019, 2024; De Bruyckere et al. 2015; Eghbali et al. 2016, 2018; Stefanini, Rendon, and Zucchelli 2020; Zucchelli et al. 2013, 2018), seven of them were classified as having low risk of bias (Roccuzzo et al. 2014, 2019, 2024; De Bruyckere et al. 2015; Eghbali et al. 2018; Zucchelli et al. 2013, 2018), while one was assigned a moderate risk of bias (Tavelli, Zucchelli, et al. 2023), and two were considered to have a high risk of bias (Stefanini, Rendon, and Zucchelli 2020; Eghbali et al. 2016).

## 4 Discussion

### 4.1 Main Findings

The present systematic review aimed to assess the available evidence on the treatment of soft tissue defects, including lack of KMW, insufficient STT, and BSTD, around single loaded/uncovered implants in the anterior area. A total of 13 studies were identified from January 2013 to February 2024. In terms of coverage of BSTD, eight articles met the inclusion criteria, whereas five reported on STT augmentation. Interestingly, none of the included studies reported on KMW augmentation.

#### 4.1.1 KMW

Despite the fact that patients were more satisfied with the esthetic appearance of implants with keratinized mucosa (Bonino et al. 2018), no studies investigating the augmentation of KMW in the esthetic area were found. On the basis of this, it has been suggested that the ideal time point to increase the width of attached keratinized mucosa is before implant placement (Thoma et al. 2022). This approach will improve the quality of soft tissues and simplify the subsequent therapeutic steps. Additionally, having a wide band of keratinized tissue reduces the risk of wound dehiscence, particularly in cases involving bone augmentation. Moreover, according to the systematic review by Basetti et al. (2016), it was recommended to address the lack of KMW during the second‐stage surgery. Taken together, these two findings may explain the lack of studies matching our inclusion criteria, as publications reporting on intervention before or during second‐stage surgery were excluded.

It is worth mentioning that an apically positioned flap in combination with FGG is considered the technique of choice for increasing KMW around implants (Sanz et al. 2022; Moraschini et al. 2022; Tavelli et al. 2021; Zucchelli et al. 2020). Nevertheless, this technique is associated with poor esthetic outcome due to color mismatch with the surrounding tissue (Zucchelli et al. 2020). These dissimilarities between the grafted areas and the adjacent regions were still visible after 5 years (Schmitt et al. 2016).

Therefore, in esthetically relevant areas, FGG should be avoided (Bassetti et al. 2016; Schmitt et al. 2016). Alternatively, CTG in combination with an apically positioned flap has been proposed to avoid the patch‐like appearance of FGG (Sanz et al. 2009). However, both techniques are associated with high patient morbidity derived from the second surgical site (Zucchelli et al. 2020). To overcome the aforementioned disadvantages of autogenous soft tissue grafts, various soft tissue substitutes have been suggested and evaluated in the scientific literature (Moraschini et al. 2022; Tavelli et al. 2021; Thoma, Strauss, et al. 2023; Montero et al. 2022). Among these, the xenogeneic collagen matrix (XCM) has demonstrated comparable results to CTG in terms of KMW augmentation and improved esthetic satisfaction (Moraschini et al. 2022; Montero et al. 2022). Recently, a prospective case series presented a new technique to reconstruct the keratinized mucosa in the esthetic area (Urban et al. 2020). This approach consisted of an apically placed strip labial gingival graft (LGG) in combination with XCM positioned coronal to the graft. The authors concluded that LGG + XCM is a valid technique to increase KMW and is associated with higher esthetic results, patient satisfaction, and low morbidity outcomes. A more recent RCT comparing this approach to FGG for the augmentation of KMW supported and confirmed these initial findings (Farooqui et al. 2023). It is worth noting that in these studies, the interventions were carried out before second‐stage surgery. Therefore, further studies are needed to confirm these findings regarding restored implants.

#### 4.1.2 BSTD

BSTDs are not a rare finding in the esthetic zone, and they negatively affect esthetics and patient satisfaction of implant therapy (Bonino et al. 2018; Tavelli et al. 2022).

The present systematic review revealed that soft tissue augmentation procedures resulted in mean BSTD coverage ranging from 28% to 99.2% (Anderson et al. 2014; Zucchelli et al. 2018). CAF, with or without vertical releasing incision, either with CTG or soft tissue substitutes, was investigated in all the included studies. This is not surprising given the fact that, after a decade of research and development of new procedures, CAF with SCTG is still considered the gold standard technique for recession coverage around teeth (Buti et al. 2013; Chambrone, Barootchi, et al. 2022; Chambrone, Botelho, et al. 2022).

In the absence of CAL loss on the adjacent teeth, CAF + CTG with previous implant crown removal and abutment reduction were associated with the highest mean BSTD coverage and complete BSTD coverage (Zucchelli et al. 2013, 2018), compared to CAF + CTG without crown removal (Roccuzzo et al. 2014, 2019, 2024; Anderson et al. 2014; Tavelli, Majzoub, et al. 2023). This superiority was related to the proliferation of the papillae following crown removal, which provided better blood supply to the CTG and the overlying flap. Moreover, improved access for interdental soft tissue de‐epithelialization was allowed and better adaptation between the graft and the reduced abutment was provided (Zucchelli et al. 2018). The influence of the restorative emergence profile contour on the gingival margin level has been underscored (González‐Martín et al. 2020). Demonstrating the efficacy of the prosthetic phase in managing BSTD, a single case report showcased this benefit (Schoenbaum et al. 2019). In this particular report, the authors have replaced the existing restoration with an interim crown. This provisional crown exhibited an undercontoured facial emergence profile and was screwed to an undermined abutment. After 3 months, the soft tissue margin migrated coronally, and the BSTD was spontaneously reduced by 1 mm. However, the prosthetic phase requires several interventions, which increases the cost and lengthens the overall treatment time (Roccuzzo et al. 2014; Tavelli, Majzoub, et al. 2023).

When the crown was retained, CAF + CTG resulted in satisfactory outcomes (Roccuzzo et al. 2014, 2019; Tavelli, Majzoub, et al. 2023; Roccuzzo et al. 2024). Despite both using split‐thickness envelop CAF in combination with de‐epithelialized FGG, Roccuzzo et al (Roccuzzo et al. 2014, 2019, 2024) achieved slightly lower BSTD coverage compared to Tavelli et al (Tavelli, Majzoub, et al. 2023). One possible explanation for this difference could be attributed to the variations in donor sites. While Tavelli, Majzoub, et al. (2023) harvested the CTG from the palate, Roccuzzo et al (Roccuzzo et al. 2014, 2019, 2024) opted to use the tuberosity area as the donor site. To the best of our knowledge, the only available data comparing these two sites around implants is derived from the RCT conducted by Rojo et al (Rojo et al. 2020). In this study, the authors found no significant differences between the two sites. However, caution should be exercised when interpreting these findings, as this RCT investigated soft tissue volume augmentation using SCTG, and the result may not apply to BSTD coverage with de‐epithelialized FGG.

The TUN had recently gained increased popularity among clinicians for root coverage due to the preservation of the integrity of the papillae, enhanced graft nutrition, and improved esthetic outcomes (Tavelli et al. 2018; Toledano‐Osorio et al. 2022; González‐Febles et al. 2023). TUN + CTG presented in the two‐arm RCT resulted in significantly lower mean BSTD coverage with 59.76% compared to CAF + CTG with 90.23% (Tavelli, Majzoub, et al. 2023). This may be due to the fact that, in the presence of a single recession, flap mobility is limited when performing TUN (Zuhr et al. 2014), which is a critical factor for recession coverage (Chambrone and Pini Prato 2019; de Sanctis and Clementini 2014). A recent systematic review and meta‐analysis has confirmed this speculation. When comparing TUN to CAF for multiple gingival recessions, similar mean root coverage was found; however, CAF significantly outperformed TUN when treating single gingival recessions (Toledano‐Osorio et al. 2022). Another possible explanation is that the authors used a modified TUN, with full‐thickness flap preparation, which may further limit flap advancement and blood supply to the underlying CTG (Tavelli et al. 2018).

To overcome issues associated with higher morbidity when autogenous CTG is used, soft tissue substitutes have been introduced. In the RCT by Anderson et al. (2014), ADM resulted in significantly lower mean BSTD coverage after 6 months (28%) compared to SCTG (40%), suggesting the superiority of autogenous SCTG for BSTD coverage around implants. However, this result should be interpreted with caution due to the small sample size, the short follow‐up, and the high risk of bias in this study.

In the presence of papilla loss and adjacent teeth exhibiting interproximal attachment loss, Tavelli, Zucchelli, et al. (2023) showed that promising results, with mean BSTD coverage of 85%, could be predictably achieved using vertical soft tissue augmentation with submerged healing. Additionally, CAL gain at the adjacent teeth and papilla reconstruction were reported. However, while thin vertical soft tissue is associated with marginal bone loss due to the establishment of biological width (Puisys and Linkevicius 2015; Linkevicius et al. 2015), excessive vertical soft tissue results in deeper probing depths and may be a risk factor for peri‐implant disease (Zhang et al. 2020). Therefore, the risk/benefit ratio of leaving the implant submerged or restoring it should be analyzed for each case separately.

When it comes to KMW gain, it seems that CTG, when used in combination with CAF or TUN, promotes KMW gain around implants. While TUN was superior to CAF in terms of KMW gain around teeth (Toledano‐Osorio et al. 2022; Gobbato et al. 2016), Tavelli et al. found significantly greater KMW increase for the CAF group (2.57 mm) compared to the TUN group (1.57 mm) (Tavelli, Majzoub, et al. 2023).

STT is a component of the peri‐implant soft tissue phenotype that has an important impact on the esthetic results (Thoma et al. 2018; Avila‐Ortiz et al. 2020). All studies reporting STT changes found a significant increase in mucosal thickness, using CTG or ADM, after the BSTD coverage procedure (Anderson et al. 2014; Tavelli, Majzoub, et al. 2023; Tavelli, Zucchelli, et al. 2023; Zucchelli et al. 2013, 2018). Anderson et al. (2014) reported the lowest STT gain following 6 months of performing ADM + CAF. A recent systematic review supported this finding, concluding that CTG demonstrated substantial STT gain when compared to soft tissue substitutes (Valles et al. 2022).

Esthetic evaluations were professionally conducted in five studies using PES/WES, CEI, and IDES (De Bruyckere et al. 2015; Anderson et al. 2014; Roccuzzo et al. 2019; Stefanini, Rendon, and Zucchelli 2020; Tavelli, Majzoub, et al. 2023). Consequently, a direct comparison between the results could not be achieved. Nevertheless, all the studies reported a significant improvement in esthetic outcomes.

A recent consensus report from the International Team for Implantology suggested that PROMs should be documented in every clinical study investigating the use of dental implants (Feine et al. 2018). Significant improvements in patients' esthetic perception, with comparable VAS scores were reported by Zuchelli et al. (2013, 2018), Roccuzzo et al. (2019, 2024), and Tavelli, Zucchelli, et al. (2023). In their RCT, TTavelli, Majzoub, et al. (2023) found substantial improvement in both groups; however, higher results were reported by the CAF group in compared to the TUN group. This is probably due to the lowest mean BSTD coverage achieved for this group. Interestingly, despite the professionally reported improvement, Anderson et al. (2014) found no change over time in either group or between groups in terms of patients' esthetic assessment. A correlation between subjective and objective esthetic evaluation has been investigated, and it has been found that professional rating is not always in accordance with patient's perception (Cosyn et al. 2017; Stefanini et al. 2018).

Regarding morbidity, the ADM group exhibited a prolonged duration of pain perception compared to the CTG group. In contrast, Stefanini et al. (2021) did not find significant differences in patient morbidity and painkiller usage between autogenous grafts and substitutes in their systematic review. A more recent systematic review and meta‐analysis arrived at a different conclusion, suggesting that soft tissue substitutes, when compared to autogenous grafts, may lead to reduced pain perception and lower consumption of painkillers following soft tissue augmentation at implant sites (Thoma, Strauss, et al. 2023). These divergent findings underscore the necessity for additional studies to achieve a comprehensive understanding of the subject.

QoL was superior in the CAF group of the RCT by Tavelli, Majzoub, et al. (2023). This is probably associated with the higher BSTD coverage observed in this group. Additionally, Anderson et al. (2014) assessed wound healing and found that the CTG group experienced a less eventful healing process compared to the ADM group. This observation could potentially explain the higher morbidity reported by the ADM group. Moreover, the higher quantity and strength of medication utilized in the CTG group can be attributed to the existence of a second surgical site in this group as compared to the ADM group. Tavelli, Zucchelli, et al. (2023) evaluated the willingness for retreatment and found that all the treated subjects would be available for retreatment if needed. This is not surprising considering the high mean BSTD coverage, good esthetic outcome, and low morbidity achieved by the intervention.

#### 4.1.3 STT

STT plays a major role not only in the esthetic outcomes (Bienz et al. 2022) but also in peri‐implant health (Thoma et al. 2018).

Our results revealed that, after a healing period of 12 months, the highest mean STT (1.2 mm) gain was achieved by Stefanini, Rendon, and Zucchelli (2020) using split thickness CAF + ADM. This successfully aligns with the findings of a systematic review by Tavelli et al. (2021), which concluded that a bilaminar approach in combination with either CTG or ADM resulted in the highest amount of STT gain. In a more recent systematic review and meta‐analysis, Yaghini et al. (2023) found no statistically significant difference in STT augmentation between ADM and autogenous grafts, suggesting the potential substitution of ADM for autogenous tissues in soft tissue augmentation around dental implants. In contrast, lower mean STT gains (0.97 and 0.83 mm) were obtained by De Bruyckere et al. (2015) and Eghbali et al. (2016), respectively, using a split‐thickness envelope + CTG after 9 months. This finding is in contrast with the available data in the literature. In fact, when comparing CTG to soft tissue substitutes in terms of STT augmentation, several systematic reviews proved the superiority of CTG, suggesting that it should be considered the gold standard grafting material (Moraschini et al. 2022; Tavelli et al. 2021; Valles et al. 2022). One possible explanation could be the difference in the surgical technique (CAF vs. envelope). Nevertheless, due to the small sample size, low quality (non‐RCT), and high heterogeneity of the aforementioned studies (follow‐up, intervention, STT recording), no conclusions can be drawn.

The long‐term outcomes of STT augmentation using CTG present conflicting findings. Sterne et al. (2016) found that 85% of the immediate post‐operative gain remained horizontally stable after 5 years. In contrast, Hosseini et al. reported that STT thickness did not undergo significant changes between baseline and 5 years. Although strong evidence of the long‐term stability of augmented soft tissues around implants is missing (Rotundo et al. 2015), the data regarding the long‐term outcome of CTG are controversial. Zucchelli et al. (2018) found a mean STT increase of 1.8 mm after 5 years of BSTD coverage, with a further increase of 0.3 mm between 1‐ and 5‐year observations. According to the author, the graft thickness was responsible for the STT gain after 1 year, while graft maturation was responsible for further STT increase after 5 years. A recent RCT comparing volume‐stable collagen matrix (VCMX) with CTG for soft tissue augmentation at implant sites found a comparable nonsignificant increase of the STT by 0.3 mm after 5 years (Thoma, Gasser, et al. 2023). Bienz et al. (2017), in a retrospective case–control study, reported a mean STT loss of 0.5 mm in both the CTG and control groups after 5 years. The variability in registration methods, timing of the augmentation procedure, and surgical techniques might be a reason for these conflicting outcomes.

Interestingly, Hosseini, Worsaae, and Gotfredsen (2020) evaluated the soft tissue level and noted a significant incisal gain of marginal soft tissue in the CTG group. Thus, despite the fact that horizontal STT remained unchanged between baseline and 5 years, the vertical mucosal level improved significantly. This finding is consistent with the results reported in a systematic review and meta‐analysis conducted by Raghoebar et al. (2021), which investigated the outcomes of soft tissue augmentation, in terms of change in vertical mucosal level and thickness at implant sites in the esthetic zone. The authors revealed that following delayed implant placement, the vertical mucosal level favored the use of grafts versus no grafts. However, the STT did not show a preference for the use of grafts compared to no grafts. This incisal migration of the mucosal margin after soft tissue grafting has been widely documented around teeth and is commonly referred to as “creeping attachment” (Matter 1980). Despite its well‐known occurrence, the underlying mechanism and predictability of this phenomenon remained unexplained (Rotundo et al. 2015; Wan et al. 2020). Creeping attachment has also been described after soft tissue augmentation around dental implants (Parra and Capri 2018; Pereira Neto et al. 2014). Thus, the observed increase in both mean and complete BSTD coverage, as reported by Zucchelli et al. (2018) and Roccuzzo et al. (2019), between 1 and 5 years, could be explained by this phenomenon.

Regarding KMW gain, Stefanini, Rendon, and Zucchelli (2020) reported an increase of 0.63 mm in the KMW after 1 year. In contrast, Hosseini, Worsaae, and Gotfredsen (2020) did not find any significant alteration in KMW between different observations or between the CTG group and control group. The role of CTG in inducing keratinization of the overlying alveolar mucosa around implants has been explored in a recent experimental study conducted in beagle dogs (Liñares et al. 2022). The authors found that the use of CTG does not induce the keratinization of the overlying mucosa. This is consistent with a recent systematic review and meta‐analysis conducted by Tavelli et al. (2021). In this review, the authors concluded that regardless of the soft tissue grafting material employed, bilaminar approaches were not associated with a significant increase in KMW. One possible explanation for the reported KMW gain by Stefanini et al. could be attributed to the surgical procedure employed. Indeed, the increase of KMW after CAF (with or without CTG) around teeth is well documented in the literature and was explained by the tendency of the mucogingival line to reestablish its genetically predetermined position (Zucchelli et al. 2014; Ainamo et al. 1992).

Concerning professional esthetic assessment, Eghbali et al. (2018) found a mean PES of 11.17 after 5 years, with no significant change compared to the 9‐month score (PES = 11). This finding is not surprising given the fact that alveolar process deficiency is the most common esthetic complication of PES (Cosyn et al. 2013; Fürhauser et al. 2017). This finding further supports the improved esthetic outcome after contour augmentation using CTG. In the CCT conducted by Hosseini, Worsaae, and Gotfredsen (2020), the papilla index scores were not significantly different between the test and control groups. However, the discoloration scores were significantly lower in the test group. This observation suggests that although CTG did not increase the thickness of peri‐implant soft tissue, it could enhance the tissue's quality, thereby improving the esthetic outcome.

### 4.2 Limitations

To the best of our knowledge, this is the first systematic review to investigate the management of soft tissue defects around osseointegrated/uncovered single implants in the esthetic area. Nevertheless, the present review does have some limitations that should be acknowledged.

First, it is important to draw attention to the few studies that were included and the fact that only two of the 12 investigations were RCTs, one of which had a high risk of bias. This is to be expected since the investigated topic is an emerging area of interest in the field of implant dentistry, and preliminary studies constitute most of the available evidence.

Second, another significant shortcoming of our review is the methodological discrepancies across studies, such as sample sizes, intervention protocols, follow‐up periods, outcome measures, and indices used. The heterogeneity of the included material precluded comparisons between studies and the drawing of conclusive statements.

Third, a limitation of most of the included studies, which by extension might be a limitation to this review, is the lack of assessment of PROMs. Fourth, due to the limited data on BSTD coverage, our findings were occasionally analyzed utilizing information derived from studies on periodontal plastic surgery. This could be a drawback, bearing in mind that peri‐implant soft tissues have different composition and vascularity, resulting in distinct healing processes compared to the gingiva (Sculean, Gruber, and Bosshardt 2014). Lastly, our search was restricted to studies published in French or English and limited to the past decade. Therefore, despite the thorough search strategy employed, which included three different databases and additional hand searches, it may still be possible that some relevant literature has been inadvertently missed during the search process.

## 5 Conclusion

Within the limitations of this systematic review, it can be concluded that soft tissue augmentation procedures resulted in satisfactory outcomes, in terms of BSTD coverage and STT increase, around loaded/uncovered single implants in the esthetic area. Regarding BSTD coverage, CAF + CTG is the most effective technique in the medium and long term. The prosthetic approach, which involves crown removal and abutment reduction, appears to enhance the outcomes of CAF + CTG. Regarding STT increase, both ADM and CTG resulted in significant gain after 1 year. Nevertheless, the long‐term stability of the obtained outcomes over time remained unclear. Management of soft tissue defects with peri‐implant plastic surgery improved the patients' esthetic appearance and QoL.

Recommendations for future research

➢ RCTs investigating KMW augmentation in the esthetic area are needed.
➢ It is strongly advised to utilize the classification of PSTDs (Zucchelli et al. 2019) in order to allow comparison between studies.
➢ New RCTs that evaluate CAF + CTG with or without a prosthetic approach and assess the cost‐effectiveness of each procedure.
➢ New RCTs that compare CAF and TUN.
➢ New RCTs evaluating BSTD coverage using CTG or soft tissue substitutes, with a minimum of 1‐year follow‐up period.
➢ RCTs comparing different donor sites and different harvesting techniques for soft tissue grafting around single implants in the anterior region.
➢ RCTs investigating STT augmentation utilizing CTG or soft tissue substitutes with extended follow‐up periods.
➢ Esthetics and PROMs should be reported in every clinical trial investigating soft tissue augmentation around implants.

Recommendations for clinical practice

➢ In cases of single BSTD without interproximal CAL loss, it is recommended that clinicians opt for CAF + CTG. Additionally, if feasible, prosthesis modification should be considered before the surgical procedure.
➢ In cases of single BSTD with interproximal CAL loss, it is advised that clinicians employ vertical soft tissue augmentation along with implant submersion.
➢ In cases of contour deficiency or dark appearance of the peri‐implant mucosa, ADM or CTG could be utilized to increase the STT.

## Author Contributions

Haithem Moussa was involved in the design of the study, study registration, literature search, acquisition and interpretation of data, article preparation, initial draft, final review, and was accountable for all aspects of the work. Wafa Nasri contributed to the design of the study, critical review of the draft, helped with the writing the article, approved the final version for publication, and was accountable for the accuracy and integrity of the work. Rania Gargouri handled the acquisition and interpretation of data, article preparation, initial draft, final review, and was accountable for all aspects of the work. Afif Bouslema was responsible for the acquisition and interpretation of data, article preparation, initial draft, final review, and was accountable for all aspects of the work.

## Ethics Statement

This systematic review did not involve primary research, and therefore, ethical approval was not required.

## Consent

As this study utilized data from publicly available sources, informed consent was not applicable.

## Conflicts of Interest

The authors declare no conflicts of interest.

## Supporting information

Supporting information.

## Data Availability

The data sets generated and analyzed during the current study are derived from publicly available sources, including PubMed, Web of Science, and Cochrane Library. The list of articles included in this systematic review, along with their unique identifiers or accession numbers, is provided in the references section of this article. The full texts of the included articles can be accessed through their respective publishers or databases. Additional information regarding the search strategy, inclusion and exclusion criteria, and data extraction process is available upon request from the corresponding author. The data that support the findings of this study are derived from the articles cited in the reference section of this manuscript and the Supporting Information. All relevant data are included within the article, its references, and the Supporting Information.
