# Supplementary material for: Management of Soft Tissue Defects Around Single Implants: A Systematic Review of the Literature
Source: Clin Exp Dent Res. 2024 Nov 4;10(6):e70003. doi: 10.1002/cre2.70003 (PMC11534634; doi:10.1002/cre2.70003)
Supplement: Supplementary file 1 — Supporting information. [file CRE2-10-e70003-s001.docx]

**Supplementary Appendix**

**Management of soft tissue defects around single implants: A systematic review of the literature**

1. **Information sources and search strategy**

According to the AMSTAR 2 guidelines, at least two databases should be searched in the Systematic review/Meta-Analysis (1), but as the number of searched databases increases, greater yield and more comprehensive, accurate results can be obtained. Therefore, the following electronic databases were used: The National Library of Medicine (MEDLINE via PubMed), the Cochrane Central Register of Controlled Trials, and Web of Science (All databases: WOS, KJD, MEDLINE, RSCI, SCIELO). A detailed systematic literature search was conducted by the two reviewers (M.H. & N.W.) independently, and any disagreements were addressed by discussion with a third reviewer (G.R.).

The search strategy was primarily designed for the MEDLINE database, employing a combination of Medical Subject Headings (MeSH) terms and keywords (Tables 1, 2 and 3). It should be noted that MeSH terms specific to peri-implant soft tissue defects are not yet available. Therefore, MeSH terms from related topics were utilized as an alternative. Subsequentely, the search strategy was modified appropriately for other databases. The applied restrictions were the publication time and the language, as specified above. The search was conducted up to February 15, 2024.

The search results from the three databases were downloaded to a single library of a citation manager "Zotero software" which assisted in combining the results obtained by all the searched databases and consequently allowed to discard duplicates automatically and keep records of the global search.

To ensure a thorough screening process, the electronic search was complemented with a manual search in the following journals: Clinical Oral Implants Research, Journal of Clinical Periodontology, Journal of Periodontology, Clinical Advances in Periodontics, Journal of Periodontal Research, Journal of Esthetic and Restorative Dentistry, and Clinical Implant Dentistry and Related Research. The manual search period was up to February 30, 2024.

Additionally, reference lists and citations of the retrieved studies for full-text screening, and previous reviews in the topic of peri-implant plastic surgery were screened (2–13).

**2. Data collection and management**

- 1. **Study selection**

The two reviewers screened the titles and abstracts (if available) of the entries identified in the search, in duplicate and independently. Next, the full text version of all studies that potentially met the eligibility criteria or for which there was insufficient information in the title and abstract to make a decision were obtained. Any article considered as potentially relevant by at least one of the reviewers was included in the next screening phase. Subsequently, the full-text publications were also evaluated in duplicate and independently by the same review examiners. The examiners were calibrated with the first six full-text, consecutive publications. Any disagreement on the eligibility of the studies was resolved through open debate between both reviewers until an agreement was reached. All articles that did not meet the eligibility criteria were excluded and the reasons for exclusion were noted (Table 4).

- 1. **Data extraction**

All relevant information from the included articles was retrieved independently by the two examiners. At any stage, if disagreements occurred between reviewers, they were resolved through by a discussion with a third reviewer. Aside from the primary and secondary outcomes, the following data were retrieved:

Study characteristics such as study design, authors, year of publication, number of centers, geographic location, setting (university vs. private practice) and source of funding.

Population characteristics, age of participants, smoking habit, number of participants, treated sites (baseline/follow-up), and follow-up period.

Type of intervention and details regarding the utilized approach for the management of soft tissue defects, grafting material(s), and techniques.

If relevant information was missing, attempts were made to contact the corresponding authors to obtain the necessary data. If the attempts were not successful, and the trial did not provide any data on any of the outcomes of interest, it was excluded.

| #1 | "Mucous Membrane"[MeSH Terms] OR "Gingival Recession"[MeSH Terms] OR "Surgical Wound Dehiscence"[MeSH Terms] OR "Mouth Mucosa"[MeSH Terms] | 245,596 |
| --- | --- | --- |
| #2 | "soft tissue dehiscence*"[Title/Abstract] OR "buccal soft tissue dehiscence*"[Title/Abstract] OR "mucosal recession*" [Title/Abstract] OR "mucosal dehiscence*" [Title/Abstract] OR "soft tissue recession*"[Title/Abstract] OR "soft tissue defect*" [Title/Abstract] OR "soft tissue deficienc*"[Title/Abstract] OR "peri implant recession"[Title/Abstract] OR "midfacial recession*" [Title/Abstract] OR "volume deficienc*" [Title/Abstract] OR "volume defect*" [Title/Abstract] OR "contour deficienc*" [Title/Abstract] OR "soft tissue volume deficienc*" [Title/Abstract] OR "gingival thickness" [Title/Abstract] OR "mucosal thickness"[Title/Abstract] OR "soft tissue thickness"[Title/Abstract] OR "keratinized mucosa" [Title/Abstract] | 7,926 |
| #3 | #1 OR #2 | 252,507 |
| #4 | "dental implantation, endosseous"[MeSH Terms] OR "dental implants, single tooth"[MeSH Terms] OR "Dental Implants"[MeSH Terms] | 36,406 |
| #5 | "single implant*"[Title/Abstract] OR "dental implant*" [Title/Abstract] OR "single tooth implant*"[Title/Abstract] OR "single tooth dental implant*"[Title/Abstract] OR "dental implantation" [Title/Abstract] OR "maxillary anterior implant*" [Title/Abstract] | 23,195 |
| #6 | #4 OR #5 | 44,804 |
| #7 | "esthetics, dental"[MeSH Terms] | 16,541 |
| #8 | "esthetic zone"[Title/Abstract] OR "esthetic area*" [Title/Abstract] OR "esthetic site*" [Title/Abstract] OR "anterior maxilla" [Title/Abstract] OR "aesthetic area*" [Title/Abstract] OR "aesthetic zone*" [Title/Abstract] OR "aesthetic site*" [Title/Abstract] OR "aesthetic region*" [Title/Abstract] OR "esthetic region*" [Title/Abstract] OR "anterior region*" [Title/Abstract] | 6,853 |
| #9 | #7 OR #8 | 22,355 |
| #10 | "Vestibuloplasty"[MeSH Terms] OR "Gingivoplasty"[MeSH Terms] OR "Connective Tissue" [MeSH Terms] OR "Surgical Flaps"[MeSH Terms] OR "Acellular Dermis"[MeSH Terms] OR "guided tissue regeneration, periodontal"[MeSH Terms] OR "Treatment Outcome" [MeSH Terms] OR "Allografts"[MeSH Terms] | 1, 653,817 |
| #11 | "soft tissue graft"[Title/Abstract] OR "soft tissue augmentation" [Title/Abstract] OR "phenotype modification" [Title/Abstract] OR "connective tissue graft"[Title/Abstract] OR "subepithelial connective tissue graft"[Title/Abstract] OR "gingival autograft"[Title/Abstract] OR "free gingival graft"[Title/Abstract] OR "allograft"[Title/Abstract] OR "alloderm"[Title/Abstract] OR "mucoderm"[Title/Abstract] OR "mucograft"[Title/Abstract] OR "Acellular Dermis" [Title/Abstract] OR "acellular dermal matrix"[Title/Abstract] OR "dermal matrix allograft" [Title/Abstract] OR "xenogeneic collagen matrix" [Title/Abstract] OR "collagen matrix" [Title/Abstract] OR "collagen matrix xenograft" [Title/Abstract] OR "Collagen-based matrix" [Title/Abstract] OR "human fibroblast derived dermal substitute" [Title/Abstract] OR "enamel matrix protein" [Title/Abstract] OR "coronally advanced flap"[Title/Abstract] OR "split thickness flap"[Title/Abstract] OR "laterally positioned flap" [Title/Abstract] OR "tunnel technique*"[Title/Abstract] OR "modified coronally advanced tunnel"[Title/Abstract] OR "coronally advanced tunnel" [Title/Abstract] OR "recession coverage" [Title/Abstract] OR "soft tissue coverage" [Title/Abstract] OR "periodontal plastic surgery" [Title/Abstract] OR "mucogingival surgery" [Title/Abstract] | 2,984 |
| #12 | #10 OR #11 | 1, 654,985 |
| #13 | #3 AND #6 AND #9 AND #12 | 222 |
| #14 | #3 AND #6 AND #9 | 423 |
| #15 | #3 AND #6 AND #12 | 897 |
| #16 | #13 OR #14 OR #15 | 1,098 |
| #17 | #13 OR #14 OR #15 Filters: English, French, from 2013 - 2024 | 557 |


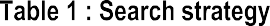

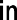

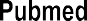

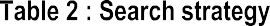

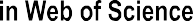


| #1 | TS=("soft tissue dehiscence*" OR "buccal soft tissue dehiscence*" OR "mucosal recession*" OR "mucosal dehiscence*" OR "soft tissue recession*" OR "soft tissue defect*" OR "soft tissue deficienc*" OR "peri implant recession" OR "midfacial recession*" OR "volume deficienc*" OR "volume defect*" OR "contour deficienc*" OR "soft tissue volume deficienc*" OR "gingival thickness" OR "mucosal thickness" OR "soft tissue thickness" OR "keratinized mucosa") | 10,152 |
| --- | --- | --- |
| #2 | TS=("single implant*" OR "dental implant*" OR "single tooth implant*" OR "single tooth dental implant*" OR "dental implantation" OR "maxillary anterior implant*") | 57,309 |
| #3 | TS=("esthetic zone" OR "esthetic area*" OR "esthetic site*" OR "anterior maxilla" OR "aesthetic area*" OR "aesthetic zone*" OR "aesthetic site*" OR "aesthetic region*" OR "esthetic region*" OR "anterior region*") | 9,069 |
| #4 | TS=("soft tissue graft" OR "soft tissue augmentation" OR "phenotype modification" OR "connective tissue graft" OR "subepithelial connective tissue graft" OR "gingival autograft" OR "free gingival graft" OR "allograft" OR "alloderm" OR "mucoderm" OR "mucograft" OR "Acellular Dermis" OR "acellular dermal matrix" OR "dermal matrix allograft" OR "xenogeneic collagen matrix" OR "collagen matrix" OR "collagen matrix xenograft" OR "Collagen-based matrix" OR "human fibroblast derived dermal substitute" OR "enamel matrix protein" OR "coronally advanced flap" OR "split thickness flap" Or "laterally positioned flap" OR "tunnel technique*" OR "modified coronally advanced tunnel" OR "coronally advanced tunnel" OR "recession coverage" OR "soft tissue coverage" OR "periodontal plastic surgery" OR "mucogingival surgery" OR "treatment outcome ") | 1,280,475 |
| #5 | #4 AND #3 AND #2 AND #1 | 163 |
| #6 | #1 AND #2 AND #3 | 267 |
| #7 | #1 AND #2 AND #4 | 559 |
| #8 | #5 OR #6 OR #7 | 663 |
| #9 | #5 OR #6 OR #7 and 2013 or 2014 or 2015 or 2016 or 2017 or 2018 or  2019 or 2020 or 2021 or 2022 or 2023 (Publication Years) and English or French (Languages) | 524 |


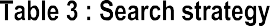

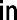

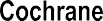

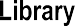


| #1 | MeSH descriptor: [Mucous Membrane] this term only | 354 |
| --- | --- | --- |
| #2 | MeSH descriptor: [Gingival Recession] this term only | 938 |
| #3 | MeSH descriptor: [Surgical Wound Dehiscence] this term only | 510 |
| #4 | MeSH descriptor: [Mouth Mucosa] this term only | 898 |
| #5 | ("soft tissue dehiscence*" OR "buccal soft tissue dehiscence*" OR "mucosal recession*" OR "mucosal dehiscence*" OR "soft tissue recession*" OR "soft tissue defect*" OR "soft tissue deficienc*" OR "peri implant recession" OR "midfacial recession*" OR "volume deficienc*" OR "volume defect*" OR "contour deficienc*" OR "soft tissue volume deficienc*" OR "gingival thickness" OR "mucosal thickness" OR "soft tissue thickness" OR "keratinized mucosa") :ti,ab,kw | 726 |
| #6 | #1 OR #2 OR #3 OR #4 OR #5 | 3221 |
| #7 | MeSH descriptor: [Dental Implantation, Endosseous] this term only | 1397 |
| #8 | MeSH descriptor: [Dental Implants, Single-Tooth] this term only | 395 |
| #9 | MeSH descriptor: [Dental Implants] this term only | 1690 |
| #10 | ("single implant*" OR "dental implant*" OR "single tooth implant*" OR "single tooth dental implant*" OR "dental implantation" OR "maxillary anterior implant*"):ti,ab,kw | 2743 |
| #11 | #7 OR #8 OR #9 OR #10 | 3390 |
| #12 | MeSH descriptor: [Esthetics, Dental] this term only | 520 |
| #13 | ("esthetic zone" OR "esthetic area*" OR "esthetic site*" OR "anterior maxilla" OR "aesthetic area*" OR "aesthetic zone*" OR "aesthetic site*" OR "aesthetic region*" OR "esthetic region*" OR "anterior region*"):ti,ab,kw | 624 |
| #14 | #12 OR #13 | 1042 |
| #15 | MeSH descriptor: [Vestibuloplasty] this term only | 42 |
| #16 | MeSH descriptor: [Gingivoplasty] this term only | 128 |
| #17 | MeSH descriptor: [Connective Tissue] this term only | 505 |
| #18 | MeSH descriptor: [Surgical Flaps] this term only | 1456 |
| #19 | MeSH descriptor: [Acellular Dermis] this term only | 97 |
| #20 | MeSH descriptor: [Guided Tissue Regeneration, Periodontal] this term only | 553 |
| #21 | MeSH descriptor: [Treatment Outcome] this term only | 169179 |
| #22 | MeSH descriptor: [Allografts] this term only | 305 |
| #23 | ("soft tissue graft" OR "soft tissue augmentation" OR "phenotype modification" OR "connective tissue graft" OR "subepithelial connective tissue graft" OR "gingival autograft" OR "free gingival graft" OR "allograft" OR "alloderm" OR "mucoderm" OR "mucograft" OR "Acellular Dermis" OR "acellular dermal matrix" OR "dermal matrix allograft" OR "xenogeneic collagen matrix" OR "collagen matrix" OR "collagen matrix xenograft" OR "Collagen-based matrix" OR "human fibroblast derived dermal substitute" OR "enamel matrix protein" OR "coronally advanced flap" OR "split thickness flap" Or "laterally positioned flap" OR "tunnel technique*" OR "modified coronally advanced tunnel" OR "coronally advanced tunnel" OR "recession coverage" OR "soft tissue coverage" OR "periodontal plastic surgery" OR "mucogingival surgery"):ti,ab,kw | 6144 |
| #24 | #15 OR #16 OR #17 OR #18 OR #19 OR #20 OR #21 OR #22 OR #23 | 175593 |
| #25 | #6 AND #11 AND #14 AND #24 | 42 |
| #26 | #6 AND #11 AND #14 | 65 |
| #27 | #6 AND #11 AND #24 | 165 |
| #28 | #25 OR #26 OR #27 with Cochrane Library publication date from Jan 2013 to  Sep 2023 | 127 |

**Table 4:** Reference and the reason for the excluded articles.

| **Reason for exclusion** | **Articles** |
| --- | --- |
| Implants not/not only in the esthetic area (n=15) | Ko et al. (14); Huang et al. (15); Vellis et al.(16) ; Esfahani et al. (17); Esfahani et al. (18); Schallhom et al. (19); Oh et al. (20); Oh et al. (21); Park et al. (22); Kikuchi et al. (23); Zafiropoulos et al.(24); Puzio et al. (25); Rojo et al. (26); Rojo et al. (27); Basegmez et al. (28);  Schmitt et al.(29) |
| Intervention before/at second stage surgery (n=7) | Tabanella. (30); Frisch et al. (31); Kobayashi et al. (32); Thoma et al. (4); Thoma et al. (33);  Thoma et al. (34); Chacón et al. (35); Bienz et al (36) |
| The effect of soft tissue surgery could not be extracted from the data (n=2) | Cosyn et al. (37); Cosyn et al. (38) |
| Histological study (n=2) | Artzi et al. (39); Sanz-Martin et al. (40) |
| Case report (n=2) | Ia et al.(41), kim et al.(42) |
| Multiple adjacent implants (n=1) | Frisch et al.(43) |
| Implant removal (n=1) | El Askari et al. (44) |
| Retrospective case series (n=1) | Le et al. (45) |

References

1. Shea BJ, Reeves BC, Wells G, Thuku M, Hamel C, Moran J, et al. AMSTAR 2: a critical appraisal tool for systematic reviews that include randomised or non-randomised studies of healthcare interventions, or both. BMJ. 21 sept 2017;358:j4008.

2. Thoma DS, Strauss FJ, Mancini L, Gasser TJW, Jung RE. Minimal invasiveness in soft tissue augmentation at dental implants: A systematic review and meta-analysis of patient-reported outcome measures. Periodontol 2000. févr 2023;91(1):182‑98.

3. Bassetti RG, Stähli A, Bassetti MA, Sculean A. Soft tissue augmentation around osseointegrated and uncovered dental implants: a systematic review. Clin Oral Investig. janv 2017;21(1):53‑70.

4. Cairo F, Barbato L, Selvaggi F, Baielli MG, Piattelli A, Chambrone L. Surgical procedures for soft tissue augmentation at implant sites. A systematic review and meta-analysis of randomized controlled trials. Clin Implant Dent Relat Res. déc 2019;21(6):1262‑70.

5. Frizzera F, Oliveira GJPL de, Shibli JA, Moraes KC de, Marcantonio EB, Marcantonio Junior E. Treatment of peri-implant soft tissue defects: a narrative review. Braz Oral Res. 2019;33(suppl 1):e073.

6. Kaddas C, Papamanoli E, Bobetsis YA. Etiology and Treatment of Peri-Implant Soft Tissue Dehiscences: A Narrative Review. Dent J (Basel). 16 mai 2022;10(5):86.

7. Kissa J, El Kholti W, Laalou Y, El Farouki M. Augmentation of keratinized gingiva around dental implants. J Stomatol Oral Maxillofac Surg. juin 2017;118(3):156‑60.

8. Mazzotti C, Stefanini M, Felice P, Bentivogli V, Mounssif I, Zucchelli G. Soft-tissue dehiscence coverage at peri-implant sites. Periodontol 2000. juin 2018;77(1):256‑72.

9. Sculean A, Chappuis V, Cosgarea R. Coverage of mucosal recessions at dental implants. Periodontol 2000. févr 2017;73(1):134‑40.

10. Soetebeer M, Jennes ME, Antonoglou GN, Al-Nawas B, Beuer F. Effectiveness of soft tissue augmentation procedures for coverage of buccal soft tissue dehiscence around dental implants: A systematic review. Clin Oral Implants Res. juin 2022;33 Suppl 23:125‑36.

11. Valles C, Vilarrasa J, Barallat L, Pascual A, Nart J. Efficacy of soft tissue augmentation procedures on tissue thickening around dental implants: A systematic review and meta-analysis. Clin Oral Implants Res. juin 2022;33 Suppl 23:72‑99.

12. Wu Q, Qu Y, Gong P, Wang T, Gong T, Man Y. Evaluation of the efficacy of keratinized mucosa augmentation techniques around dental implants: a systematic review. J Prosthet Dent. mai 2015;113(5):383‑90.

13. Zucchelli G, Tavelli L, Stefanini M, Barootchi S, Wang HL. The coronally advanced flap technique revisited: Treatment of peri-implant soft tissue dehiscences. Int J Oral Implantol (Berl). 2 nov 2021;14(4):351‑65.

14. Ko KA, Lee JS, Kim JH, Park JM, Gruber R, Thoma DS. Changes in mucogingival junction after an apically positioned flap with collagen matrix at sites with or without previous guided bone regeneration: A prospective comparative cohort study. Clin Oral Implants Res. déc 2020;31(12):1199‑206.

15. Huang JP, Liu JM, Wu YM, Dai A, Hu HJ, He FM, et al. Clinical evaluation of xenogeneic collagen matrix versus free gingival grafts for keratinized mucosa augmentation around dental implants: A randomized controlled clinical trial. J Clin Periodontol. oct 2021;48(10):1293‑301.

16. Vellis J, Kutkut A, Al-Sabbagh M. Comparison of Xenogeneic Collagen Matrix vs. Free Gingival Grafts to Increase the Zone of Keratinized Mucosa Around Functioning Implants. Implant Dent. févr 2019;28(1):20‑7.

17. Namadmalian Esfahani N, Khorsand A, Mohseni Salehimonfared S. The influence of harvesting free gingival graft on self-reported pain perception: A randomized two-arm parallel clinical trial. J Dent Sci. janv 2021;16(1):410‑6.

18. Namadmalian Esfahani N, Mohseni Salehi Monfared SH, Khorsand A, Shamshiri AR. Dimensional changes of keratinized mucosa after accordion versus conventional free gingival graft around dental implants: A randomized two-arm parallel clinical trial. Clin Oral Implants Res. mai 2022;33(5):472‑81.

19. Schallhorn RA, McClain PK, Charles A, Clem D, Newman MG. Evaluation of a porcine collagen matrix used to augment keratinized tissue and increase soft tissue thickness around existing dental implants. Int J Periodontics Restorative Dent. 2015;35(1):99‑103.

20. Oh SL, Masri RM, Williams DA, Ji C, Romberg E. Free gingival grafts for implants exhibiting lack of keratinized mucosa: a prospective controlled randomized clinical study. J Clin Periodontol. févr 2017;44(2):195‑203.

21. Oh SL, Ji C, Azad S. Free gingival grafts for implants exhibiting a lack of keratinized mucosa: Extended follow-up of a randomized controlled trial. J Clin Periodontol. juin 2020;47(6):777‑85.

22. Park WB, Kang KL, Han JY. Long-Term Clinical and Radiographic Observation of Periimplant Tissues After Autogenous Soft Tissue Grafts: A 15-Year Retrospective Study. Implant Dent. oct 2017;26(5):762‑9.

23. Kikuchi T, Wada M, Mameno T, Hasegawa D, Serino G, Ikebe K. Longitudinal study on the effect of keratinized mucosal augmentation surrounding dental implants in preventing peri-implant bone loss. PeerJ. 2022;10:e13598.

24. Zafiropoulos GG, Al-Asfour AA, Abuzayeda M, Kačarević ZP, Murray CA, Trajkovski B. Peri-Implant Mucosa Augmentation with an Acellular Collagen Matrix. Membranes (Basel). 12 sept 2021;11(9):698.

25. Puzio M, Hadzik J, Błaszczyszyn A, Gedrange T, Dominiak M. Soft tissue augmentation around dental implants with connective tissue graft (CTG) and xenogenic collagen matrix (XCM). 1-year randomized control trail. Ann Anat. juill 2020;230:151484.

26. Rojo E, Stroppa G, Sanz-Martin I, Gonzalez-Martín O, Alemany AS, Nart J. Soft tissue volume gain around dental implants using autogenous subepithelial connective tissue grafts harvested from the lateral palate or tuberosity area. A randomized controlled clinical study. J Clin Periodontol. avr 2018;45(4):495‑503.

27. Rojo E, Stroppa G, Sanz-Martin I, Gonzalez-Martín O, Nart J. Soft tissue stability around dental implants after soft tissue grafting from the lateral palate or the tuberosity area - A randomized controlled clinical study. J Clin Periodontol. juill 2020;47(7):892‑9.

28. Basegmez C, Karabuda ZC, Demirel K, Yalcin S. The comparison of acellular dermal matrix allografts with free gingival grafts in the augmentation of peri-implant attached mucosa: a randomised controlled trial. Eur J Oral Implantol. 2013;6(2):145‑52.

29. Schmitt CM, Tudor C, Kiener K, Wehrhan F, Schmitt J, Eitner S, et al. Vestibuloplasty: porcine collagen matrix versus free gingival graft: a clinical and histologic study. J Periodontol. juill 2013;84(7):914‑23.

30. Tabanella G. Buccal Pedicle Flap Technique Combined With Porcine Collagen Matrix for Volumetric Augmentation of Peri-Implant Mucosa. Clin Adv Periodontics. mars 2022;12(1):5‑11.

31. Frisch E, Ratka-Krüger P, Ziebolz D. Increasing the Width of Keratinized Mucosa in Maxillary Implant Areas Using a Split Palatal Bridge Flap: Surgical Technique and 1-Year Follow-Up. J Oral Implantol. oct 2015;41(5):e195-201.

32. Kobayashi T, Nakano T, Ono S, Matsumura A, Yamada S, Yatani H. Quantitative evaluation of connective tissue grafts on peri-implant tissue morphology in the esthetic zone: A 1-year prospective clinical study. Clin Implant Dent Relat Res. juin 2020;22(3):311‑8.

33. Thoma DS, Gasser TJW, Jung RE, Hämmerle CHF. Randomized controlled clinical trial comparing implant sites augmented with a volume-stable collagen matrix or an autogenous connective tissue graft: 3-year data after insertion of reconstructions. J Clin Periodontol. mai 2020;47(5):630‑9.

34. Thoma DS, Gasser TJW, Hämmerle CHF, Strauss FJ, Jung RE. Soft tissue augmentation with a volume-stable collagen matrix or an autogenous connective tissue graft at implant sites: Five-year results of a randomized controlled trial post implant loading. J Periodontol. févr 2023;94(2):230‑43.

35. Chacón R GJ, Retana L. The connective tissue graft as a membrane to improve esthetics according the defect. J Stomatol Oral Maxillofac Surg. oct 2022;123(5):514‑20.

36. Bienz SP, Carrera ER, Hüsler J, Roccuzzo A, Jung RE, Thoma DS. Soft tissue contour changes at implant sites with or without soft tissue grafting in the esthetic zone: A retrospective case–control study with a 12-year follow-up. Clinical Oral Implants Research. 2023;34(5):521‑30.

37. Cosyn J, De Bruyn H, Cleymaet R. Soft tissue preservation and pink aesthetics around single immediate implant restorations: a 1-year prospective study. Clin Implant Dent Relat Res. déc 2013;15(6):847‑57.

38. Cosyn J, Eghbali A, Hermans A, Vervaeke S, De Bruyn H, Cleymaet R. A 5-year prospective study on single immediate implants in the aesthetic zone. J Clin Periodontol. août 2016;43(8):702‑9.

39. Artzi Z, Renert U, Netanely E, Thoma DS, Vered M. Histologic composition of marginal mucosal tissue augmented by a resorbable volume-stable collagen matrix in soft tissue thickening procedures in humans: a morphometric observational study. Clin Oral Investig. janv 2022;26(1):427‑35.

40. Sanz-Martín I, Rojo E, Maldonado E, Stroppa G, Nart J, Sanz M. Structural and histological differences between connective tissue grafts harvested from the lateral palatal mucosa or from the tuberosity area. Clin Oral Investig. févr 2019;23(2):957‑64.

41. Urban IA, Klokkevold PR, Takei HH. Papilla Reformation at Single-Tooth Implant Sites Adjacent to Teeth with Severely Compromised Periodontal Support. Int J Periodontics Restorative Dent. 2017;37(1):9‑17.

42. Kim HJ, Lee JY, Joo JY. Surgical Approach for Facial Peri-implant Soft Tissue Deficiency in the Esthetic Area. J Implantol Appl Sci. 31 déc 2022;26(4):288‑94.

43. Frisch E, Ratka-Krüger P. A new technique for peri-implant recession treatment: Partially epithelialized connective tissue grafts. Description of the technique and preliminary results of a case series. Clin Implant Dent Relat Res. juin 2020;22(3):403‑8.

44. El Askary AES, Ghallab NA, Tan SC, Rosen PS, Shawkat A. Implant-Related Gingival Recession: Pilot Case Series Presents Novel Technique and Scoring Template. Compend Contin Educ Dent. juill 2016;37(7):468‑80.

45. Le B, Borzabadi-Farahani A, Nielsen B. Treatment of Labial Soft Tissue Recession Around Dental Implants in the Esthetic Zone Using Guided Bone Regeneration With Mineralized Allograft: A Retrospective Clinical Case Series. J Oral Maxillofac Surg. août 2016;74(8):1552‑61.
